# SifR is an Rrf2-family quinone sensor associated with catechol iron uptake in *Streptococcus pneumoniae* D39

**DOI:** 10.1016/j.jbc.2022.102046

**Published:** 2022-05-18

**Authors:** Yifan Zhang, Julia E. Martin, Katherine A. Edmonds, Malcolm E. Winkler, David P. Giedroc

**Affiliations:** 1 Department of Chemistry, Indiana University, Bloomington, Indiana, USA; 2 Department of Molecular and Cellular Biochemistry, Indiana University, Bloomington, Indiana, USA; 3 Department of Biological Sciences, Idaho State University, Pocatello, Idaho, USA; 4 Department of Biology, Indiana University, Bloomington, Indiana, USA

**Keywords:** iron homeostasis, bacterial iron metabolism, quinone sensor, transcriptional repressor, catechol, catecholamine, Adc, adrenochrome, *Bs*YwnA, *Bacillus subtilis* YwnA, BQ, 1,4-benzoquinone, C23O, catechol 2,3 dioxygenase, CatE, catechol 2,3-dioxygenase, DHBS, 2,3-dihydroxybenzylserine, DMBQ, dimethoxybenzoquinone, ESI, electrospray ionization, FAD, flavin adenine dinucleotide, Fe, iron, Fe^II^, ferrous iron, Fe^III^, ferric iron, H_2_O_2_, hydrogen peroxide, MS, mass spectrometry, NE, norepinephrine, NEM, *N*-ethylmaleimide, NTA, nitrilotriacetic acid, O/P, operator/promoter, PDB, Protein Data Bank, Pia, pneumococcal iron acquisition, Piu, pneumococcal iron uptake, qRT–PCR, quantitative RT–PCR, ROS, reactive oxygen species, S, sulfur, SifR, streptococcal IscR-like family transcriptional repressor, *Sp*SifR, *S. pneumoniae* SifR, SSN, sequence similarity network, TCEP, Tris(2-carboxyethyl)phosphine

## Abstract

*Streptococcus pneumoniae* (pneumococcus) is a Gram-positive commensal and human respiratory pathogen. How this bacterium satisfies its nutritional iron (Fe) requirement in the context of endogenously produced hydrogen peroxide is not well understood. Here, we characterize a novel virulence-associated Rrf2-family transcriptional repressor that we term SifR (streptococcal IscR-like family transcriptional repressor) encoded by *spd_1448* and conserved in *Streptococci*. Global transcriptomic analysis of a Δ*sifR* strain defines the SifR regulon as genes encoding a candidate catechol dioxygenase CatE, an uncharacterized oxidoreductase YwnB, a candidate flavin-dependent ferric reductase YhdA, a candidate heme-based ferric reductase domain–containing protein and the Piu (pneumococcus iron uptake) Fe transporter (*piuBCDA*). Previous work established that membrane-anchored PiuA binds Fe^III^–*bis*-catechol or monocatechol complexes with high affinity, including the human catecholamine stress hormone, norepinephrine. We demonstrate that SifR senses quinone *via* a single conserved cysteine that represses its regulon when in the reduced form. Upon reaction with catechol-derived quinones, we show that SifR dissociates from the DNA leading to regulon derepression, allowing the pneumococcus to access a catechol-derived source of Fe while minimizing reactive electrophile stress induced by quinones. Consistent with this model, we show that CatE is an Fe^II^-dependent 2,3-catechol dioxygenase with broad substrate specificity, YwnB is an NAD(P)H-dependent quinone reductase capable of reducing the oxidized and cyclized norepinephrine, adrenochrome, and YhdA is capable of reducing a number of Fe^III^ complexes, including PiuA-binding transport substrates. These findings are consistent with a model where Fe^III^–catechol complexes serve as significant nutritional Fe sources in the host.

*Streptococcus pneumoniae* (*S. pneumoniae*; pneumococcus) is a low-GC Gram-positive aerotolerant anaerobe that is naturally competent and highly genetically adaptable. *S. pneumoniae* is a common commensal resident of the human upper respiratory tract, where it colonizes epithelial mucosal surfaces of the host nasopharynx asymptomatically as part of a diverse microbial community (1). Myriad physiological signals, from both bacterial and host origins, including stress (2), trigger an incompletely understood transition of *S. pneumoniae* into a life-threatening invasive pathogen that can propagate in the middle ear, causing acute otitis, the lower respiratory tract, and the lung, causing pneumonia, the blood stream, causing bacteremia, and the brain meninges, causing meningitis (1, 3, 4). *S. pneumoniae* causes significant mortality annually worldwide and has become increasingly resistant to antibiotics (5).

Bacterial virulence factors aid transition of *S. pneumoniae* from a commensal to an invasive organism by adapting or evading the host immune and inflammatory responses (1). Among the strongest virulence factors is iron (Fe) acquisition by *S. pneumoniae*. Early studies establish that *S. pneumoniae* strains lacking both Fe^III^-uptake ABC-transporter systems, Pia (pneumococcal iron acquisition) and Piu (pneumococcal iron uptake), are strongly attenuated for virulence in pulmonary and systemic infection murine models (6). The combination of PiuA and PiaA soluble binding proteins induces protection against systemic *S. pneumoniae* infections in mice and thus were considered as early vaccine candidates (7). Note that Fe uptake is a virulence determinant for nearly all bacterial pathogens, and this is the foundational basis of “nutritional immunity,” in which the infected host restricts Fe and other critical transition metals from invading pathogens (8, 9, 10, 11). As such, successful pathogens have evolved numerous nonoverlapping strategies to acquire both ferric iron (Fe^III^) as solubilized Fe^III^ chelates and ferrous iron (Fe^II^) from the infected host to meet nutritional Fe requirements (12, 13, 14).

In *S. pneumoniae*, Fe must be efficiently managed as a result of its unusual physiology. *S. pneumoniae* is a fermentative lactic acid bacterium that derives all its energy needs from anaerobic glycolysis and the associated pyruvate node of aerobic metabolism, which interconverts lactate and acetyl phosphate through pyruvate, *via* the action of the two enzymes, lactate oxidase (LctO) and pyruvate oxidase (SpxB) (15). Both enzymes utilize O_2_ as a substrate and release hydrogen peroxide (H_2_O_2_), a toxic reactive oxygen species (ROS), as a byproduct; this is the primary mode of respiration by the *S. pneumoniae* since the organism lacks the tricarboxylic acid cycle and respiratory electron transfer chain. Access to acetyl phosphate allows substrate-level phosphorylation of ADP by acetate kinase to make a third molecule of ATP (16). The absence of a tricarboxylic acid cycle and an electron transfer chain significantly reduces the cellular quota of Fe-requiring enzymes. A survey of predicted Fe–sulfur (S) proteins revealed just 11 enzymes in *S. pneumoniae* compared with ≈140 in *Escherichia coli*; most of which are expected to function under strict anaerobic conditions (17). The quota of heme and nonheme Fe enzymes in *Spn* is not well understood. As such, *S. pneumoniae* is considered a “manganese-centric” organism that accumulates approximately equal total concentrations of Fe and Mn when cultured in rich growth medium (18, 19).

Fe homeostasis in *S. pneumoniae* is regulated by the orphan response regulatory RitR (20, 21, 22), which regulates the expression of *piu* genes, but does so by not responding to reversible Fe^II^ binding. Instead, RitR employs a single redox-sensitive Cys, C128, the oxidation state of which is reported to modulate RitR DNA-binding activity (23). Under conditions of low ROS, RitR exists as a reduced monomeric protein that binds weakly to the DNA operator allowing for constitutive expression of *piuBCDA* (23). As ROS levels rise, RitR forms a number of oxidative forms, one of which is a disulfide-crosslinked dimer that binds more tightly to the DNA-triggering repression of *piu* expression and Fe uptake (23). RitR deletion strains suffer from Fe toxicity that can be rescued by exogenous addition of manganese (20).

In previous work, we reclassified the *S. pneumoniae* PiuBCDA transporter and in particular, the ligand-binding component of this ABC transporter PiuA, from a heme transporter as had been commonly assumed (6, 24) to a transporter that is specific for coordinatively unsaturated Fe^III^–catecholate complexes (25, 26). *S. pneumoniae* PiuA is structurally and functionally similar to *Campylobacter jejuni* CeuE and *Staphylococcus aureus* SstD, each of which bind and transport tetracoordinate Fe^III^–catecholate complexes using two protein-derived ligands to complete the octahedral coordination complex around the Fe^III^ (25, 27, 28, 29). All three transporters can bind either 2 mol eq of a monocatechol or a single mole equivalent of a *bis*-catechol (25, 27). Both *S. aureus* SstD and *S. pneumoniae* PiuA bind Fe^III^ complexes of the host-derived catecholamine stress hormone norepinephrine (NE), and in the case of *S. aureus*, this contributes to its bacterial virulence (29). Both *S. aureus* SstD and *S. pneumoniae* PiuA can liberate and capture Fe^III^ from host transferrin in the presence of O_2_, which endows these organisms the ability to scavenge Fe from important host sources that are generally employed by the host to restrict access to this essential micronutrient. We postulated that this chemistry may well be a critical feature in the transition of *S. pneumoniae* from a commensal to an invasive pathogen, since NE has been shown to increase the growth and migration of *S. pneumoniae* to the lungs (2, 30). This process is strongly impacted by PiuA, RitR, and Fe binding properties of NE, as well as other bacterial factors (2, 30, 31).

We reasoned that in order to effectively utilize Fe^III^–NE complexes as nutritional sources of Fe during invasive disease, *S. pneumoniae* would have to avoid the toxicity associated with accumulated *bis*-hydroxy catechols, which would spontaneously autooxidize to the semiquinone radical and the quinone species in the presence of ambient O_2_ and H_2_O_2_. Quinones are potent reactive electrophile species that react with abundant cellular nucleophiles, including the extra cyclic amines of DNA bases and amines and thiolates of the proteome (32). This motivated a search for an uncharacterized transcriptional regulator that, like *piu*, was a documented virulence factor and that could be connected to Fe regulation or a reactive electrophile species response. This led us to the protein encoded by *spd_1448* in *S. pneumoniae* serotype 2 D39. SPD_1448 is an Rrf2-family transcriptional repressor (33) that is ubiquitous among streptococci and entirely uncharacterized. In this study, we rename *spd_1448* encoding SPD_1448 to SifR (streptococcal IscR-like family transcriptional repressor), define the SifR regulon, and demonstrate that SifR is a monothiolate quinone sensor. This activity contrasts sharply with IscR, an Fe–S-containing regulator that senses Fe–S cluster status in *E. coli*, or the myriad of nitric oxide sensors that allow adaptation of bacteria to reactive nitrogen species *via* Fe–S cluster decomposition (34, 35, 36). Using genomic enzymology tools, we place SifR in the context of the Rrf2 superfamily of transcriptional repressors and present a biochemical characterization of nearly all identified key SifR-regulated gene products. Our data taken collectively are consistent with a regulatory model where SifR senses cellular quinones, thus allowing bacterial cells to utilize simple host-abundant Fe^III^–catecholamine complexes that are taken up through the PiuBCDA transporter as a nutritional Fe source, all while avoiding collateral quinone toxicity (37).

## Results

### *S. pneumoniae* SifR is a novel Rrf2-family repressor that harbors a single conserved cysteine

**Figure 1:**
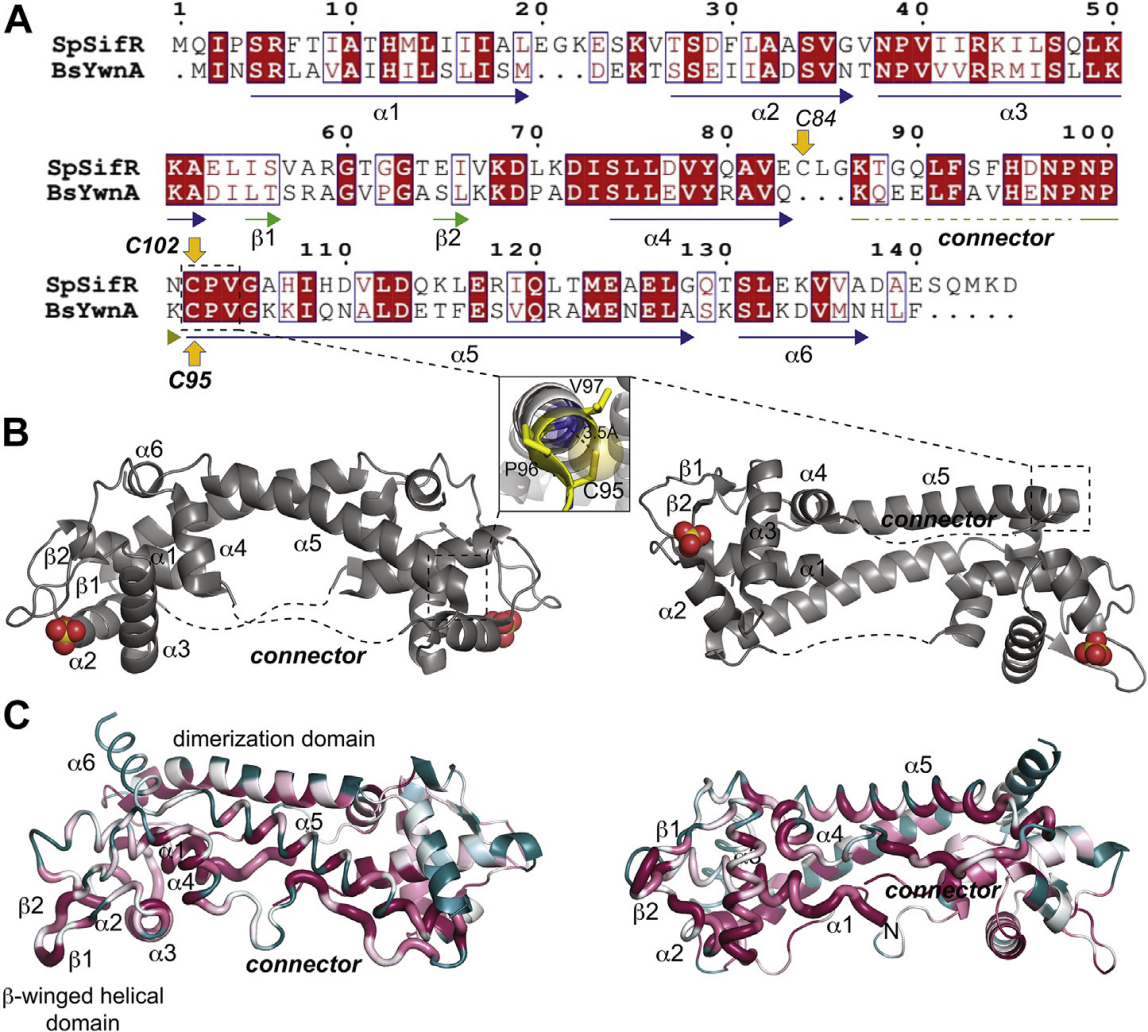
**Sequence alignment and structural models of *Bacillus subtilis* YwnA (*Bs*YwnA) and *Sp*SifR.***A*, sequence alignment of *Sp*SifR and *B. subtilis* YwnA (locus tag: BSU36680) showing the secondary structure of YwnA (PDB code: 1XD7). The Cys in *Sp*SifR is indicated with the conserved Cys in *Sp*SifR and *Bs*YwnA highlighted. *B*, ribbon representations of the structure of *B*sYwnA (*left*, side view; *right*, DNA-binding face) with the secondary structures and connector region highlighted for the one protomer. *Inset*, close-up of the CPV (Cys95-Pro96-Val97) region at the N-terminal end of the α5 helix, with a N-capping H-bond shown. *C*, Alphafold2 (68, 92) model of *Sp*SifR in putty representation, colored by residue conservation determined with ConSurf (93) (*maroon* = conserved, *cyan* = variable), with one subunit in *ribbon* representation and the other in *sausage* representation with thickness corresponding to sequence conservation (*thick*, high conservation). The β-winged helical domains, connector, and the dimerization domains are indicated. PDB, Protein Data Bank; *Sp*SifR, *Streptococcus pneumoniae* SifR.

Initial investigations of the literature suggest that *S. pneumoniae spd_1448* (renamed *sifR* here) encodes an Rrf2-type family transcriptional regulatory protein we now term SifR. The genomic neighborhood of *sifR* provided no clues as to the function of SifR, except that the *sifR* gene is transcribed from the opposite strand upstream and relatively adjacent to *spd_1450*, which encodes a Mn^II^-sensing metalloregulatory protein PsaR (38, 39). Functionally characterized members of the Rrf2 family fall into two general classes: (1) those that harbor an atypical 4Fe–4S or 2Fe–2S cluster that senses oxidative or nitrosative stress at the Fe–S site (35) and (2) those not known to harbor a cluster but contain a pair of Cys residues (40). The prototypical Rrf2-family repressor is proteobacterial IscR, an Fe–S cluster regulator that contains a 2Fe–2S cluster ligated by three Cys and one His and controls the biogenesis of Fe–S clusters in cells (34, 41, 42). A sequence alignment reveals that SifR shares 42% identity and 63% similarity to *Bacillus subtilis* YwnA (*Bs*YwnA), encoded by *ywnA* as part of the *ywnAB* operon (Fig. 1*A*). Although the structure of *B. subtilis* YwnA is known (Protein Data Bank [PDB] code: 1XD7; Fig. 1*B*) and its expression is induced by exogenous catechol (43), its function is unknown. *S. pneumoniae* SifR (*Sp*SifR) and *Bs*YwnA would appear to represent a third major class of Rrf2-family repressor that harbors a single conserved cysteine (C102 in *Sp*SifR in Fig. 1*A*) as documented below.

In general, Rrf2 repressors are dimers consisting of ≈150 residue subunits, with an N-terminal DNA-binding “winged helical” domain connected to a C-terminal helical domain by a ≈20-residue region of irregular secondary structure, as shown in *B*sYwnA (Fig. 1*B*). The DNA-binding domain contains the α1 helix followed by an irregular loop, the α2–α3 helix–turn–helix motif followed by the β1–β2 wing, which often contains an RGxxGG “wing-tip,” and terminates with the α4 helix. The α4 helix is followed by long “connector” that links the winged helical domain with the α5 helix, which forms an antiparallel α5–α5′ coiled coil that provides much of the dimerization interface; this is followed by a variable-length α6 helix. The N-terminal region of an α5 helix from one subunit packs against the winged helical domain of the opposite subunit within the homodimer (Fig. 1*B*). In the case of IscR, metal ligands are found in the C-terminal region of the “connector” and residues in the α5 helix generally conforming to a Cys-X_5_-Cys-X_5_-Cys-X_2_-His sequence (41). In other Fe–S cluster–containing Rrf2 repressors, metal ligands are shared between this connector and the N-terminal α1 helix of the opposite subunit (35).

**Figure 2:**
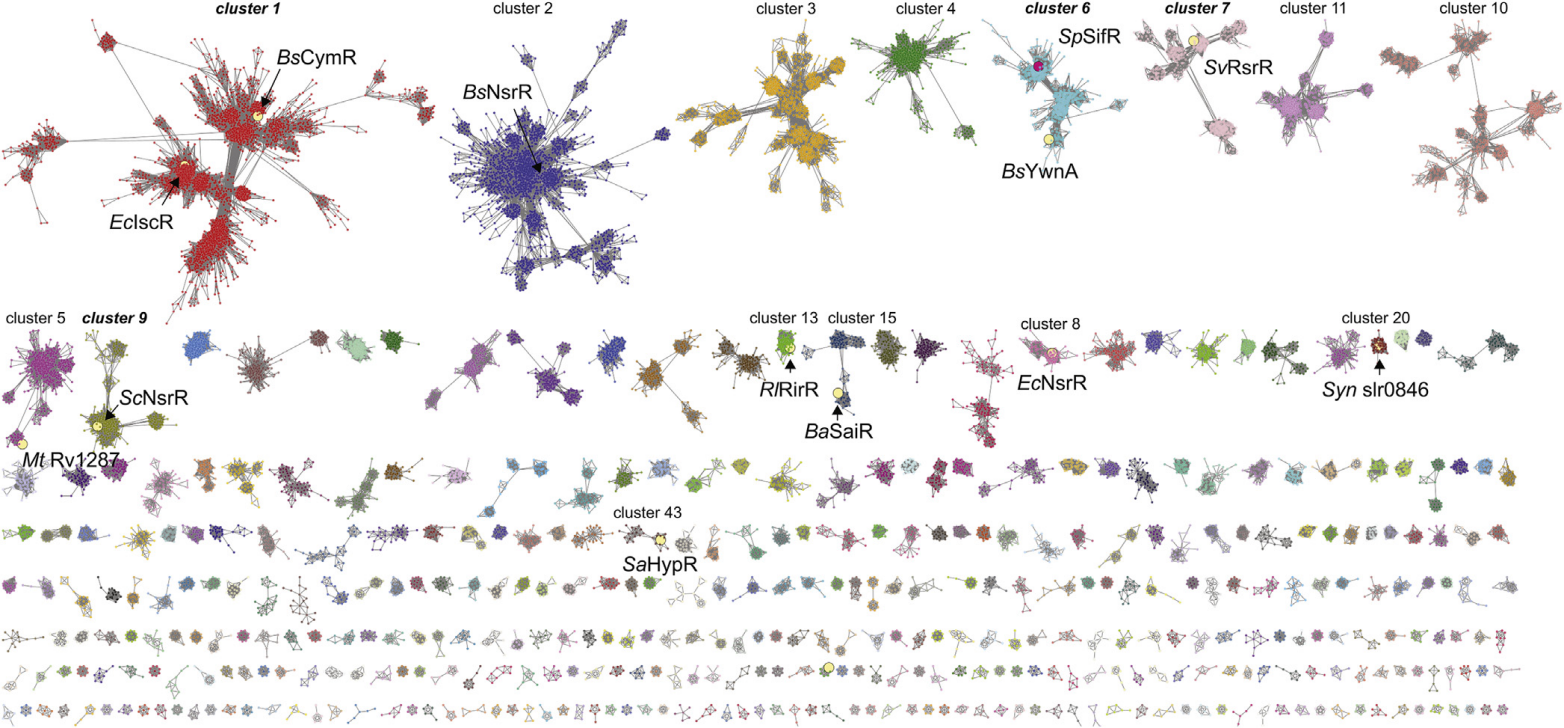
**Sequence similarity network (SSN) analysis of Rrf2 superfamily of transcriptional regulators using SPD_1448 as query (InterPro Family: IPR000944) with an alignment score of 43.** SSN clusters with greater than seven metanodes are shown and ranked according to the number of unique sequences in each SSN cluster (with one being the largest number of sequences) and arranged from *upper left* to *lower right* by decreasing numbers of sequence nodes (each node contains sequences that are 80% identical over 90% of the sequence). SSN clusters for which there is a biochemically characterized, functionally characterized, or a SwissProt-validated member (indicated by the *yellow circle*) are highlighted by “cluster #,” and those containing validated members of known structure are further highlighted by “cluster #.” SSN cluster 6 (node cluster rank 5) harbors *Sp*SifR characterized in this work. See text for additional details and Tables S1 and S2 for a complete list of all clusters, singletons, and associated UniProt identifiers in this database. *Bs*, *Bacillus subtilis*; *Ec*, *Escherichia coli*; *Mt*, *Mycobacterium tuberculosis*; *Rl*, *Rhizobium leguminoserum*; *Sa*, *Staphylococcus aureus*; *Sc*, *Streptomyces coelicolor*; *Sv*, *Streptomyces venezuelae*; *Syn*, *Synechocystis*.

To obtain detailed insights into amino acid sequence conservation of *Sp*SifR and place SifR in the context of other Rrf2-family regulators, we subjected SifR to a sequence similarity network (SSN) analysis (Fig. 2) (44, 45). We carried out these analyses using the *Sp*SifR sequence and the corresponding InterPro Family (IPR000944) as query. To analyze the retrieved sequences, we first used an alignment score of 26 to group those sequences sharing ≥40% identity over 80% of the sequence into a single SSN cluster. This constraint allows *Sp*SifR and *Bs*YwnA to colocalize on a 50% representative node (repnode50) map (Fig. S1). All known characterized Fe–S cluster-harboring Rrf2 repressors are found in SSN cluster 1 and comprise 80.3% of all unique sequences in nonsingleton clusters (see below). SSN cluster 2 corresponds to 15.9% of all such sequences and includes *Sp*SifR and *Bs*YwnA (Fig. S1). All sequences in the SSN cluster 2 harbor a single conserved cysteine and are thus representative of a large subfamily of monothiolate Rrf2 repressors not yet characterized.

In an effort to further segregate these sequences into isofunctional SSN clusters, we analyzed these data with an alignment score of 43 (where proteins with ≈50% identity over 80% of the sequence roughly group into single clusters). Further restraining the sequence length to 100 to 200 residues resulted in 1852 SSN clusters and 2306 singletons harboring 25,852 metanodes (with 80% sequence identity over 80% of the sequence) (Fig. 2). IscR and CymR, the master regulator of cysteine biosynthesis in *S. aureus* and *B. subtilis* (46, 47), are found in distinct subclusters of SSN cluster 1 and comprise ≈20% of all sequences (Fig. S2). The next largest grouping of Fe–S cluster–containing regulators is the global nitric oxide stress response regulator NsrR, which harbors a subunit-bridging 4Fe–4S center coordinated by D8, C93, C98, and C105 in the *Streptomyces coelicolor* sequence (48) (Fig. S3*A*). Characterized NsrRs from *S. coelicolor*, *E. coli*, and *B. subtilis* are found in SSN clusters 9, 8, and 2, respectively (Fig. 2). SSN cluster 13 consists of a group of regulators that harbor a labile 4Fe–4S cluster of unknown structure and includes the Fe-responsive regulator RirA found in plant symbionts (Rhizobia ssp.) and pathogens; a WebLogo plot of sequence conservation reveals four invariant Cys residues in a ligating arrangement reminiscent of NsrR (Fig. S3*A*). Redox sensor RsrRs are grouped in SSN cluster 7. RsrRs harbor a highly unusual subunit bridging 2Fe–2S cluster that reversibly cycles between +2 and +1 oxidation states, the latter of which binds weakly to DNA, and thus is a sensor of cellular redox status (49, 50). In the *Streptomyces venezuelae* RsrR, the 2Fe–2S cluster is coordinated by the highly conserved residues E8, H12, C90, and C110, with W9 mediating the allosteric redox switch (Fig. S3*A*) (49). Finally, this SSN analysis reveals three large SSN clusters 3, 4, and 10, which account for 10.1% of all sequences (Fig. S2) that have distinct patterns of conserved residues but remain uncharacterized (Fig. S3*B*). Furthermore, two large SSN clusters 5 and 20 associated with mycobacterial and cyanobacterial species, respectively, appear to lack conserved Cys and His residues but do retain a number of aromatic residues (Tyr/Trp) in key regulatory positions discusssed above (Fig. S3*B*).

Two functionally characterized dithiol Rrf2-family regulators include the redox sensors SaiR from *Bacillus anthracis* (cluster 15) (40) and HypR from *S. aureus* (cluster 43) (Fig. 2) (51). SaiR conserves two Cys arranged in C-X_7_-C motif in the C-terminal region of the connector, whereas HypR conserves two Cys widely spaced in the sequence (Fig. S3*C*). The Cys in *Sa*HypR (C33 and C99) engages in reversible disulfide bond formation in response to the potent oxidant, hypochlorite, during host infection (51). Finally, this SSN analysis reveals that *Bs*YwnA and *Sp*SifR segregate into subclusters within SSN cluster 6 and as discussed previously are characterized by a single conserved Cys residue in the *N*-cap position of the α5 helix as part of a conserved HxxPNPx**C** sequence (Fig. 1*B*, *inset*; Figs. S1 and S3*C*). Cluster 6 sequences comprise 2.3% of all Rrf2 sequences examined (Fig. S2) and are the subject of the work presented here.

### Candidate SifR-regulated genes are involved in Fe and catechol/quinone metabolism

**Figure 3:**
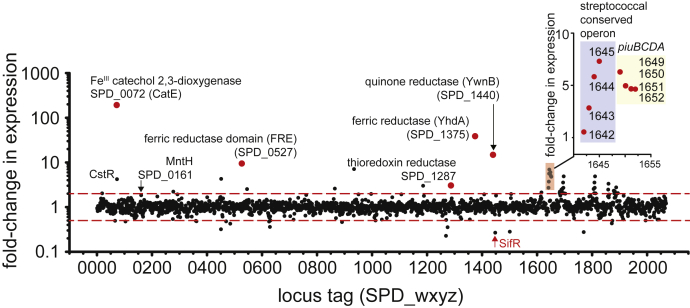
**RNA-Seq analysis of differential transcription in the Δ*sifR* (*spd_1448*) *Streptococcus pneumoniae* D39 strain relative to isogenic WT strain under anaerobic conditions.** Genes of interest are indicated by their presumptive functions or common name and locus tag protein designations. For a complete list of differentially expressed genes and all genes detected, see Table S5.

Working from the hypothesis that SifR is a transcriptional repressor like other Rrf2-family members, we constructed a *sifR*-null deletion in encapsulated *S. pneumoniae* D39W (Δ*sifR*) (Tables S4 and S5). WT and Δ*sifR* strains were grown in rich medium under strict anaerobic conditions. We chose anaerobic conditions so as to reduce any ROS stress interference, since production of endogenous H_2_O_2_ is limited under these conditions. This allowed us to focus on the impact of Δ*sifR* on *S. pneumoniae* growth and global gene expression by RNA-Seq (Table S5). Comparison of WT *versus* Δ*sifR* strains reveals a handful of genes with differential expression at least twofold that are candidate SifR regulatory targets (Fig. 3). The most strongly differentially expressed gene (191-fold) is *spd_0072* that encodes an uncharacterized metal-dependent catechol 2,3-dioxygenase, termed CatE (52). Two NAD(P)H-dependent oxidoreductases were also identified. The first oxidoreductase, YwnB (SPD_1440), has a homolog in *B. subtilis* that is encoded in the same operon as the YwnA candidate catechol sensor (43). The structure of pneumococcal YwnB is known (locus tag SP_1627 in *S. pneumoniae* TIGR4; PDB code: 4R01), but its function is not (see below). The second oxidoreductase, YhdA (SPD_1375), which has a homolog in *B. subtilis*, is a strong candidate for a ferric (Fe^III^ to Fe^II^) (53) or quinone reductase (54, 55). An uncharacterized integral membrane protein and putative diheme, extracytoplasmic reducing ferric (FRE) domain protein (*spd_0527*) (56, 57, 58) is also a likely SifR target since it is upregulated in the Δ*sifR* mutant. The sole thioredoxin reductase (*trxB*; *spd_1287*) is increased approximately threefold in the Δ*sifR* strain relative to WT, an extent similar to that of the persulfide sensor CstR (*spd_0073*) (59). The repression of selected SifR-regulated genes in a *sifR* strain that was repaired with a WT *sifR* allele strain is recovered as measured by quantitative RT–PCR (qRT–PCR) (Fig. S4*A*).

We note that expression of the high-affinity tetradentate catechol–Fe^III^ transporter and known RitR target *piuBCDA* is increased in expression in the Δ*sifR* strain (21, 23, 60). This suggests that SifR-regulated genes may serve an important role in allowing access specifically to catechol-derived Fe^III^ sources. qRT–PCR was used next to confirm differential expression of select genes found by RNA-Seq analysis, while also exploring if SifR is an active repressor under microaerophilic conditions, where endogenous H_2_O_2_ levels can reach upward of 100 μM (17). These gene expression data are broadly consistent with the conclusions reached by RNA-Seq carried out under strict anaerobic growth conditions (Fig. S4*B*). These data suggest that SifR is an active repressor even under conditions of endogenous H_2_O_2_ production (17) and must sense something other than endogenous H_2_O_2_, as described later.

### SifR binds to a canonical Rrf2 DNA operator upstream of SifR-regulated genes

**Figure 4:**
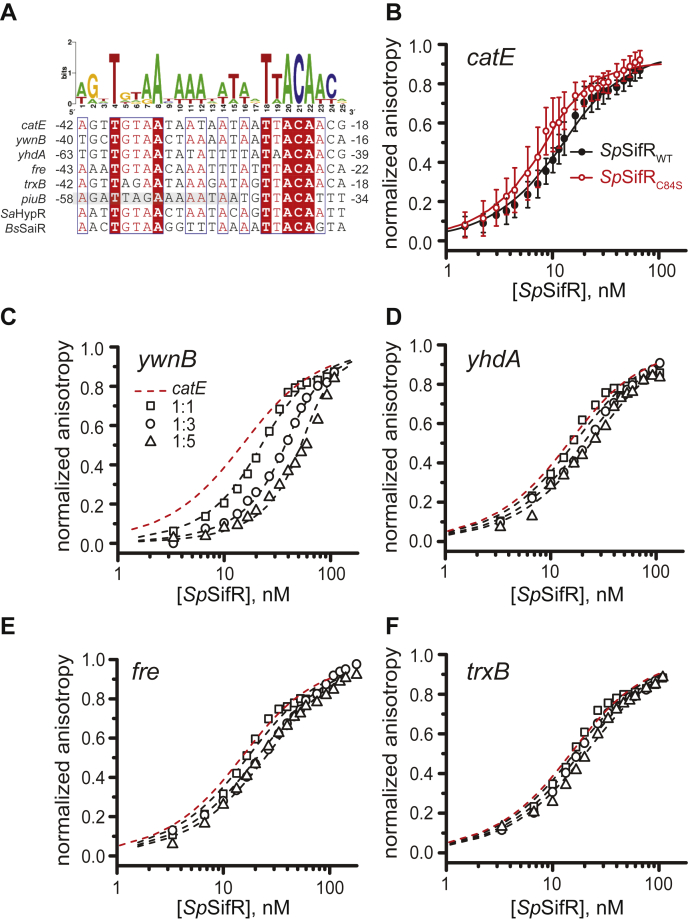
**Binding of *Sp*SifR to DNA operator–promoters (O/Ps) of candidate SifR-regulated genes.***A*, candidate SifR O/P regions upstream of the genes encoding *catE (spd_0072)*, *ywnB (spd_1440)*, *yhdA* (*spd_1375*), *fre* (*spd_0527*), *trxB* (*spd_1287*), and *piuB* (*spd_1649*, with partial RitR-binding site, shaded) compared with DNA operators for Rrf2 repressors *Sa*HypR and *Bs*SaiR. A consensus operator is shown at the *top*. *B*, *catE* O/P binding by WT (*filled black circles*) *versus* C84S (*filled red circles*) SifR to a fluorescein (F)-labeled DNA duplex, F-*catE* O/P. The continuous lines through the data represent fits to a 1:1 binding model using DynaFit (91), with the error bars representative of the SD of triplicate datasets. *C*–*F*, DNA binding to the F-*catE* O/P (*red dashed line*) in the presence of equimolar (1:1; *open squares*, *C*–*F*), 3:1 (*open circles*; *C*–*F*), and 5:1 (*open triangles*, *C*–*F*) of unlabeled (*C*) *ywnB*, (*D*) *yhdA*, (*E*) *fre*, and (*F*) *trxB* O/P-containing duplexes. The continuous lines through the data are global fits of all three datasets to a competition binding model (Fig. S10), with the *K*_*a*_ (*catE*) fixed the value determined in *B* using DynaFit (91) with these parameters compiled in Table 1. Conditions: 25 mM Tris–HCl, 150 mM NaCl, 2 mM EDTA, 2 mM TCEP (pH 7.5), 25.0 °C. CatE, catechol 2,3-dioxygenase; *Sp*SifR, *Streptococcus pneumoniae* SifR; TCEP, Tris(2-carboxyethyl)phosphine.

To identify SifR-regulated genes, we searched for an approximately palindromic Rrf2-like DNA operator upstream of candidate-regulated genes that possess similarity to the core palindromic TGTAA-x_9_-TTACA motif known to bind HOCl sensor *S. aureus* HypR (cluster 43; Fig. 2). This was motivated by the uniquely high pairwise sequence similarity of the α3 or reading head helix of the helix–turn–helix motif in SifR *versus* HypR (Fig. S3*C*). This led to the identification of a 19-bp near-palindromic operator sequence, TGTAA-N_9-_TTACA (Fig. 4*A*). We then prepared dsDNA duplexes of 31 to 33 bps in the length with the DNA operator placed approximately in the middle of its native genomic context (Table S4) and measured SifR DNA-binding affinities using a quantitative fluorescence anisotropy–based method (Fig. 4). We attached a fluorescein probe to one end of a DNA duplex encompassing the *catE* (*spd_0072*) DNA operator and titrated in reduced WT or C84S SifR mutant protein (Fig. 4*B*). The resulting data fit to single nondissociable homodimer-binding model, which extracted a *K*_*a*_ of ≈10^8^ M^−1^ under our conditions (Table 1; 25 mM Tris–HCl, 150 mM NaCl, 2 mM EDTA, 2 mM Tris(2-carboxyethyl)phosphine [TCEP], pH 7.5, 25 °C). We note that the WT, C84S, C102S, and C84S/C102S mutants are all homodimers by analytical gel-filtration chromatography (Fig. S5); however, any substitution of the conserved C102 results in nonspecific binding of SifR to the DNA or severe aggregation on the DNA (see later), thereby preventing a quantitative analysis of these data (Fig. S8*B*). We find that WT SifR binds tightly to the nearly perfectly symmetric *catE* operator, whereas the C84S SifR mutant binds with equal or greater affinity, thus revealing that nonconserved C84 (Figs. 1*A* and S3) is not required for DNA recognition.

**Table 1:** DNA-binding affinities of WT *S. pneumoniae* SifR for duplexes harboring DNA O/P sequences^a^

Locus tag or gene name O/P	*K*_*a*_ (×10^8^) (M^−1^)^b^
*catE (spd_0072)*	WT: 1.0 (±0.2); C84S: 4.8 (±1.5)
*ywnB* (*spd_1440*)	3.2 (±0.4)
*yhdA* (*spd_1375*)	0.23 (±0.04)
*fre* (*spd_0527*)	0.25 (±0.05)
*trxB* (*spd_1275*)	0.13 (±0.04)

a Sequence of the core operator regions shown in Figure 4A (see Table S4 for complete sequences of the dsDNAs used here), with DNA-binding data shown in Figure 4.

b Upper limit for this competition assay under these conditions is 0.1 × 10^8^ M^−1^. Conditions: 25 mM Tris–HCl, 150 mM NaCl, pH 7.4, 25.0 °C.

We then carried out the same anisotropy-based titrations with WT SifR and *catE* operator DNA, but in the presence of an equimolar, threefold or fivefold molar excess of an unlabeled duplex, which harbors a distinct DNA operator (Fig. 4, *C*–*F*). These competition-binding isotherms were then globally analyzed to obtain *K*_*a*_ for all other DNA operators tested (Table 1). These experiments reveal a hierarchy of DNA-binding affinities that tracks roughly with differential gene expression (Fig. 3) and the degree to which the pseudopalindromic operator tends toward near perfect twofold symmetry (Fig. 4*A*). We find that the SifR binds most tightly to *catE* and *ywnB* operators (log *K*_*a*_ ≈ 8.3), followed by *yhdA* and *fre* operators (log *K*_*a*_ ≈ 7.4), and *trxB* (log *K*_*a*_ ≈ 7.1). These experiments establish that the four most highly differentially expressed genes (*catE*, *ywnB*, *yhdA*, and *fre*) in the Δ*sifR* strain are direct SifR targets, whereas *trxB* remains only a potential target possibly because it has essentially one half-site (Fig. 4*A*). Interestingly, the SifR-binding site mapped upstream of *piuBCDA* also contains a half-site with three substitutions in the downstream TGTAA sequence, which partially overlaps one of the RitR-binding sites (Fig. 4*A*). The functional significance is that this finding is unknown but suggests the possibility that RitR and SifR collaborate or alternatively differentially regulate *piuBCDA* expression.

### Spd_0072 encodes a broad spectrum Fe^II^-dependent catechol 2,3 dioxygenase

**Figure 5:**
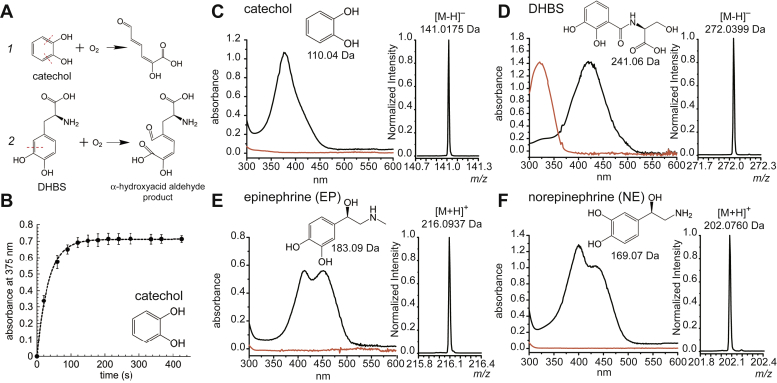
**Product analysis of *Sp*CatE-catalyzed reactions.***A*, schematic representation of CatE-catalyzed O_2_-dependent C–C bond cleavage in (1) catechol and (2) DHBS (*red dashed lines*). *B*, kinetics of catechol cleavage from triplicate experiments, with the *solid line* a fit to single exponential, *k* = 0.029 ± 0.001 s^−1^. Error bars represent the SD of triplicate data. *C*–*F*, electronic absorption spectra (*red*, substrate; *black*, product) and mass spectrometry analysis of the products of CatE-catalyzed cleavage for catechol (*C*), DHBS (*D*), epinephrine (*E*), and NE (*F*). The observed masses of the substrates and products are shown as measured by negative ion mode (*C* and *D*) and positive ion mode (*E* and *F*). Conditions: 5 μM Fe^II^–CatE, 100 μM indicated catechol, pH 7.4, ambient temperature, all 5 min reactions. CatE, catechol 2,3-dioxygenase; DHBS, 2,3-dihydroxybenzylserine; NE, norepinephrine.

Sequence analysis suggests that *spd_0072* encodes a catechol 2,3 dioxygenase or CatE, a well-studied enzyme that generally functions in the catabolism of aerobic aromatic compounds (43, 52, 61). Catechol dioxygenases open the catechol aromatic ring *via* either *ortho* (intradiol) or *meta* (extradiol) cleavage, catalyzed by a Fe^III^-dependent catechol 1,2 dioxygenase or an Fe^II^- or Mn^II^-dependent catechol 2,3 dioxygenase (C23O), respectively (Fig. 5*A*). The ring-opened semialdehyde products are then further integrated into bacterial metabolism. We purified SPD_0072 to homogeneity, and after loading with equimolar Fe^II^ under anaerobic conditions and verified by inductively coupled plasma mass spectrometry (MS), we first monitored its activity using UV–Vis spectroscopy against freshly prepared catechol in the presence of ambient O_2_ at pH 7.4, initiating the reaction with the enzyme (Fig. 5*B*). An absorption peak at 375 nm appears within 20 s with the reaction reaching at plateau after several minutes. This change in absorbance is indicative of the production of 2-hydroxymuconate semialdehyde (62), with the mass of the product consistent with extradiol cleavage of the substrate and incorporation of two oxygen atoms (Δ*m* = 31 Da for the [M–H]^–^ ion) (Fig. 5*C* and Table S8). Only the Fe(II)-reconstituted enzyme is active, with no activity observed with Mn(II) (data not shown). These experiments confirm that *spd_0072* encodes an authentic C23O, and we therefore, rename this enzyme CatE (catechol extradiol dioxygenase) (52).

As *S. pneumoniae* is unlikely to encounter catechol during an infection, we evaluated the activity of *Sp*CatE against a number of other monocatechols and *bis*-catechols, including two hydrolysis products of the *tris*-catecholate siderophore enterobactin, 2,3-dihydroxybenzylserine (DHBS) and the DHBS-dimer (data not shown), and a number of host-derived catecholamines, including NE, epinephrine, and l-dihydroxyphenylalanine (Fig. S6) using an end point (5 min) assay (Fig. 5, *D*–*F*). We note that the Fe^III^–catecholate transporter PiuA forms high-affinity complexes with NE, DHBS, and di-DHBS, and thus, these may be bioavailable in the pneumococcal cell (25); further, degradation of bacillibactin (a catecholate Fe^III^-siderophore) in *B. subtilis* is known to involve a C23O and is important in catechol recycling (52). We found that the *Sp*CatE can utilize each of these compounds as substrates, with the exact masses of the products verified by MS (Fig. 5, *C*–*F*, *insets* and Table S8). Since the catechol “side chain” is *ortho* to one of the hydroxy substituents in DHBS and *meta* in the catecholamines, this suggests that *Sp*CatE has rather broad substrate specificity and may well cleave the unencumbered side of the dihydroxy substituent.

### Spd_1440 (YwnB) encodes a versatile NAD(P)H-dependent quinone reductase

**Figure 6:**
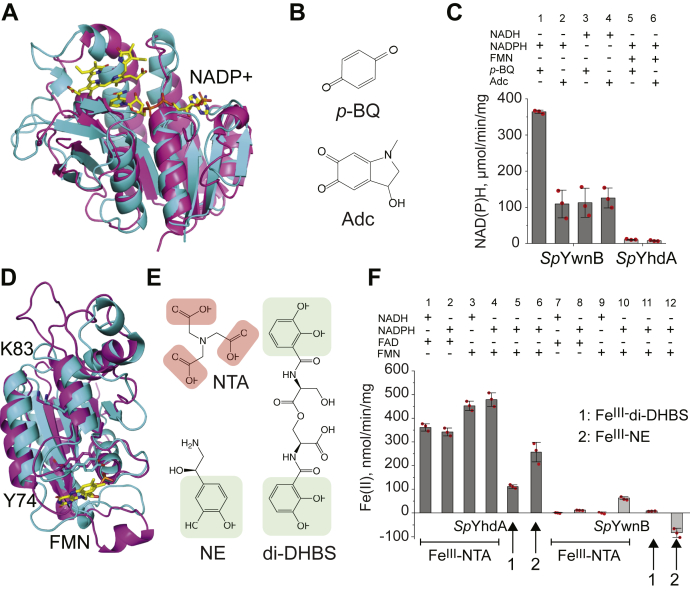
***Sp*YwnB is a NAD(P)H-dependent quinone reductase, whereas *Sp*YhdA is a NAD(P)H:flavin dependent Fe**^**III**^**-reductase.***A*, ribbon representation of *Sp*YwnB (PDB code: 4R01, *cyan*), overlaid with human biliverdin IXβ reductase (PDB code: 1HE3, *magenta*), in complex with NADP cofactor and substrate (*yellow*), indicating likely binding sites for cofactor and substrate in *Sp*YwnB. *B*, chemical structures of YwnB substrates used in quinone reductase assay: 1,4-benzoquinone (*p*-BQ) and adrenochrome (Adc) shown in *C*. *C*, the results of triplicate end-point assays (2 min) of quinone reductase activity of *Sp*YwnB (10 nM) or *Sp*YhdA (250 nM) with 100 μM *p*-BQ or 100 μM Adc as electron acceptors as indicated, in the presence of 100 μM NAD(P)H. About 10 μM FMN was added to the *Sp*YhdA assays. *D*, ribbon representation of an Alphafold2 model of SPD_1375 (*cyan*) overlaid on the structure of *Bs*YhdA (*magenta*; PDB code: 2GSW) with FMN (*yellow*) and two active-site residues, Y74 and K83 (in the α3 helix) indicated in *stick*. *E*, chemical structures of Fe^III^-chelating molecules used in the ferric reductase activity assays (F): nitrilotriacetic acid (NTA), norepinephrine (NE), and linear dimer of dihydroxybenzoylserine (di-DHBS). *F*, the results of triplicate end-point assays of ferric reductase activity of *Sp*YhdA (0.25 μM) and *Sp*YwnB (0.25 μM) using 100 μM Fe^III^–NTA, 50 μM Fe^III^–di-DHBS, or 50 μM Fe^III^–NE as electron acceptors as indicated and 100 μM NADH or NADPH in the presence of 10 μM FAD or FMN. About 500 μM ferrozine was present to capture Fe^II^. Error bars in *C* and *F* represent the SD of triplicate measurements, with each data point shown (*red circles*). PDB, Protein Data Bank.

The genes encoding YwnA and YwnB are adjacent in many bacterial genomes, although not in *S. pneumoniae*. The structure of *Sp*YwnB has been determined (PDB code: 4R01; SP1627 from *S. pneumoniae* TIGR4, identical to SPD_1440) and shows an α/β dinucleotide binding fold, similar to that of human biliverdin IXβ reductase, which catalyzes the NAD(P)H-dependent reduction of a range of biliverdin, flavin, pyrroloquinoline quinine, and ferric ion substrates (Fig. 6*A*) (63). We therefore hypothesized that YwnB is likely a pyridine nucleotide-dependent quinone reductase, given that SifR senses quinones (see later) (64). Purified *Sp*YwnB is colorless upon purification, consistent with a lack of a tightly bound cofactor. We tested both quinone reductase and flavin-dependent ferric reductase activities and found that YwnB is active against both a model 1,4-benzoquinone (*p*-BQ) as well as adrenochrome (Adc; Fig. 6*C*), derived from the spontaneous 2-e^–^ oxidation and cyclization of epinephrine (Fig. 6*B*). In contrast, *Sp*YhdA has detectable, but much lower, activity in this assay under these conditions (Fig. 6*C*).

### Spd_1375 (YhdA) encodes an authentic NAD(P)H-dependent FMN ferric reductase

Sequence similarity suggests that SPD_1375 is a flavoprotein and homolog of a thermostable *B. subtilis* NADPH:FMN azo-, Fe^III^, and quinone reductase, YhdA. Another *Bs*YhdA homolog from *Saccharomyces cerevisiae* is also reported to possess ferric reductase activity (65), analogous to that observed in other flavin-requiring NAD(P)H oxidoreductases that can access the semiquinone (1e^–^ reduced) radical (53, 66, 67). An AlphaFold2 (68) structural model of *Sp*YhdA closely resembles the structure of the *Streptococcus mutans* homolog (58% identical to SPD_1375; PDB code: 3FVW), which has not been biochemically characterized. However, the model is also similar to the structure of *Bs*YhdA, with two key catalytic residues, Y74 and K83, aligned around what appears to be a solvated active site (Fig. 6*D*). We therefore evaluated the ferric reductase activity of *Sp*YhdA and *Sp*YwnB using three different Fe^III^ complexes, including two catecholates, the enterobactin hydrolysis product, di-DHBS, and NE (Fig. 6*E*). We first used 100 μM Fe^III^–nitrilotriacetic acid (NTA) as a model ferric Fe substrate (69), evaluating FMN *versus* flavin adenine dinucleotide (FAD) as electron donors, and NADH *versus* NADPH as reductants (Fig. 6*F*). We find that *Sp*YhdA has significant activity and prefers FMN over FAD, like *Bs*YhdA (55), with little dependence on the nature of the pyridine nucleotide. *Sp*YhdA also has activity against the two Fe^III^–catecholate substrates. YwnB, in contrast, shows detectable activity only with FMN and NADPH with Fe^III^–NTA, albeit approximately sevenfold lower than that of YhdA under these conditions, and no activity against either catecholate–Fe^III^ complex (Fig. 6*F*). We conclude that *Sp*YwnB is an NAD(P)H-dependent quinone reductase, whereas SPD_1375 is a promiscuous NAD(P)H:FMN ferric reductase.

### SifR reacts with quinones *via* C102 leading to dissociation from the DNA

**Figure 7:**
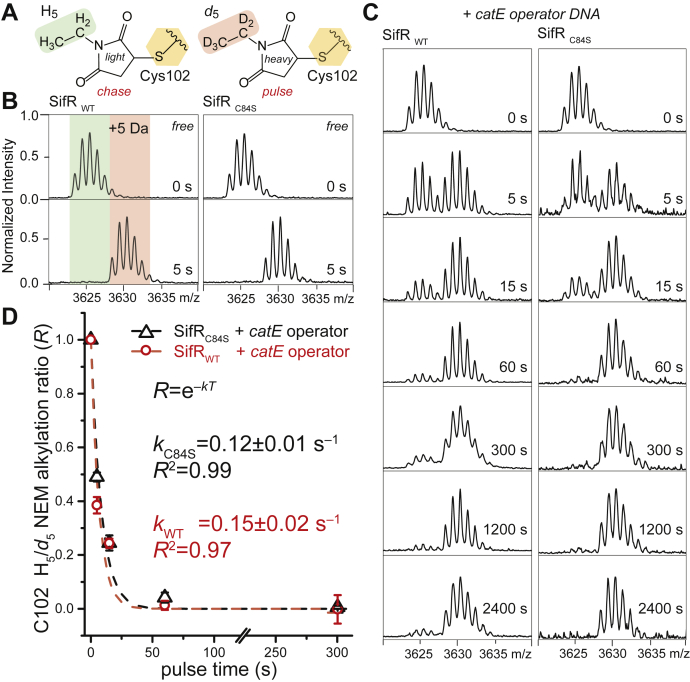
**Ratiometric pulsed alkylation-mass spectrometry analysis of C102 in *Sp*SifR free and bound to the DNA operator.***A*, schematic of the NEM molecules used in the pulse (heavy, *shaded red*, *d*_5_) and chase (light, *shaded green*, H_5_) times of the experiment (*B*) MALDI-MS analysis of the C102-containing peptide before and after a 5 s pulse with *d*_5_-NEM for the WT (*left*) and C84S SifRs (*right*). *C*, analogous to those experiments shown in *B*, but reactions were carried out with the SifR-*catE* operator-promoter containing DNA complex. *D*, kinetic analysis of the data shown in *C*, with resolved pseudo–first-order rate constants shown by the *dashed lines*. Error bars represent the SD of triplicate data. CatE, catechol 2,3-dioxygenase; NEM, *N*-ethylmaleimide.

The information presented suggests a regulatory model where SifR employs a single cysteine residue, C102, to react with catechol-derived quinones, thus allowing access to nutritional Fe^II^, while avoiding reactive electrophile stress. Indeed, the known catechol sensor in *B. subtilis*, YodB, reacts directly with a model quinone, methyl-*p*-BQ, using a conserved cysteine thiol that results in transcriptional derepression of the YodB regulon (52, 70). Many bacterial pathogens encode dedicated thiol-based quinone sensors, used to combat host-derived oxidative stressors (37). We first evaluated the intrinsic reactivity of C102 toward a neutral electrophile, *N*-ethylmaleimide (NEM), both on and off the DNA, using a pulsed-chase derivatization strategy, in which an incubation of pulse time, *t*, with heavy (*d*_5_) NEM, is followed by a chase with a large excess of light (H_5_) NEM (60, 71). Samples are then subjected to trypsin digestion and the peptides resolved by MALDI–MS (Fig. 7). These data reveal complete modification of C102 with a *d*_5_-NEM pulse time of 5 s when free (unbound) in solution, and when bound to DNA, detectable protection is observed, but with complete derivatization occurring with *d*_5_-NEM pulse time of ≈60 s (Fig. 7, *B* and *C*). Fitting these data to a first-order reaction scheme gives rise to a rate constant, *k* = 0.12 (±0.01) s^−1^, with very similar rates obtained with the WT and C84S SifR proteins bound to DNA (Fig. 7*D*). Note that tryptic peptide containing C84 is not detected in this experiment, likely because of poor ionization efficiency. The structure of *Bs*YwnA (Fig. 1*B*) suggests that the reactivity of C102 in SifR (C97 in YwnA) may be enhanced by accepting a hydrogen bond from the backbone NH of V104 (3.5 Å), which would lower the p*K*_a_ of the C102 thiolate and increase its nucleophilicity (Fig. 1*B*, *inset*).

**Figure 8:**
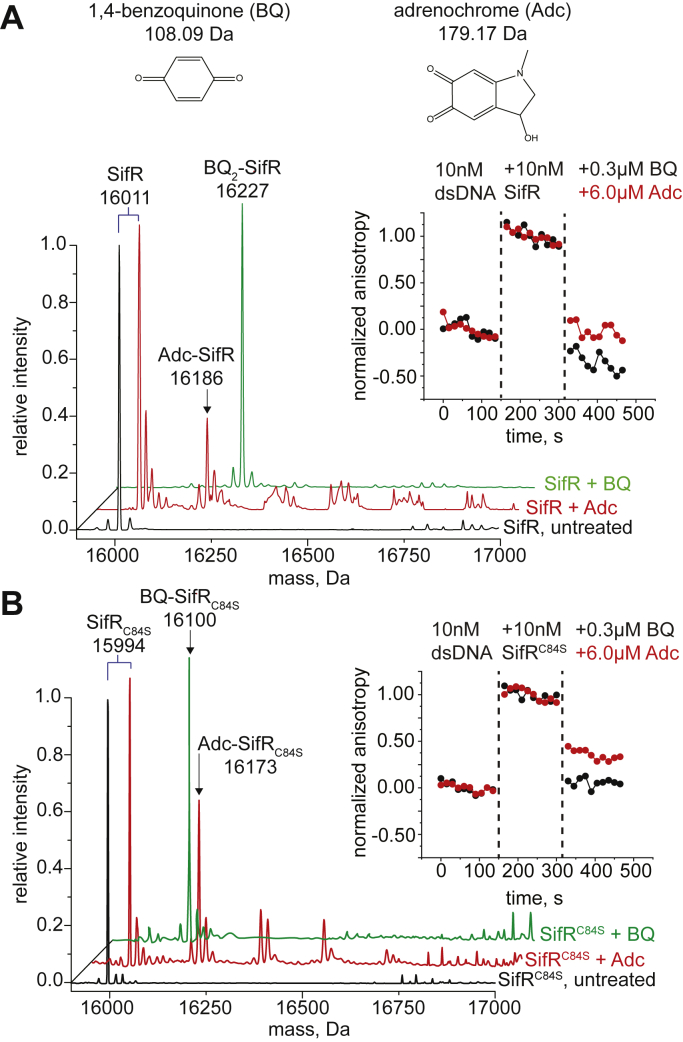
**LC–ESI–MS analysis of the products resulting from the reaction of WT (*A*) or C84S (*B*) SifR with a 20-fold thiol excess of adrenochrome (Adc; *red*) or 1,4-benozoquinone (BQ; *green*) relative to untreated protein (*black*)**. The masses of the resulting products are shown as are the masses for BQ and Adc (in *A*). Expected masses are BQ_2_-SifR, 16,227 Da; Adc-SifR, 16,190 Da; BQ-C84S SifR, 16,101 Da; and Adc-C84S SifR, 16,173 Da. Companion LC–MS/MS data of WT SifR are shown in Figure 7. *Insets*, normalized dsDNA anisotropy change induced by quinone modification of the WT (*A*) or C84S (*B*) SifR dimer. The anisotropy was monitored continuously. Proteins or quinones were added to the indicated final concentration at the time point indicated by the *vertical dashed lines*. Triplicate experiments were performed, with one representative dataset shown. ESI, electrospray ionization.

We next carried out a series of end-point reactions (1 h, pH 7.5) of WT or C84S SifR with a 20-fold excess of BQ and Adc and resolved these products by electrospray ionization (ESI)–MS, and tandem LC–ESI–MS/MS to identify the site of modification (Figs. 8 and S7). These reactions reveal that WT SifR reacts quantitatively with the BQ to yield a 2:1 adduct, whereas Adc reacts more slowly to yield some monoadducted product and a trace of doubly adducted product (Fig. 8*A* and Table S7). This reveals that the nonconserved C84 can react with electrophiles, like the sensing thiolate C102. For C84S SifR, only the monoadducted products are formed, again with BQ much more reactive (Fig. 8*B*). Tandem LC–MS/MS analysis of the WT or C84S SifR-derived C102-containing tryptic peptide is consistent with formation of a new C–S bond at C102 using both electrophiles (Fig. S7).

We next wished to establish that quinone modification of C102 in SifR was necessary and sufficient to dissociate SifR from the DNA operator. We took two approaches to do this. In the first, we simply added BQ and Adc to WT and C84S SifR–*catE* operator complexes, which results in a rapid dissociation of the complex as measured by a decreased fluorescence anisotropy indicative of weaker binding (Fig. 8, *insets*). In addition, we formed by the fully BQ-adducted WT and C84S SifRs and titrated this into a fluorescein-labeled *catE* operator DNA (Fig. S8*A*); this isotherm was significantly shifted to the right and was not saturable, indicative of weak, likely nonspecific binding. We see analogous behavior with C102S and C84S/C102S SifR proteins (Fig. S8*B*), revealing that the integrity of C102 is required for both DNA binding and allosteric inhibition of binding as a result of quinone adduction. This finding is consistent with the finding that C102S SifR is poorer repressor in cells than in cells expressing the WT or C84S SifR alleles (Fig. S9).

**Figure 9:**
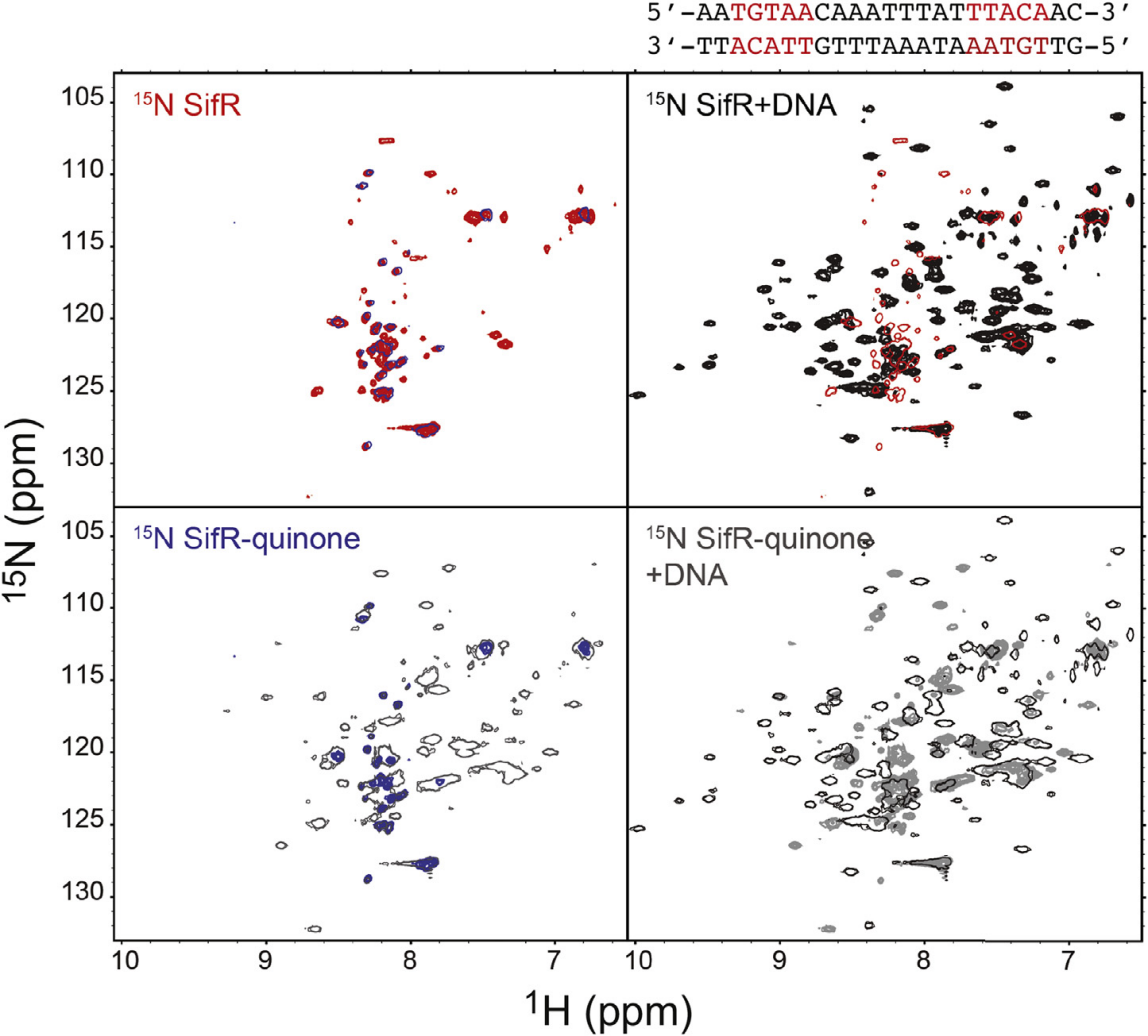
^**1**^**H,**^**15**^**N-TROSY spectra obtained for the SifR homodimer free in solution (*red* crosspeaks, *upper left*), bound to a 23-bp *fre* operator duplex (*black* crosspeaks, *upper right*), following addition of DMBQ to the DNA complex (*gray* crosspeaks, *lower right*), compared with the DMBQ adduct alone (*blue* crosspeaks, *lower left*).** In all cases, the spectrum to which the indicated spectrum is compared is its characteristic color, plotted at single contour. The 23-bp duplex containing the *fre* DNA operator is shown (*upper right*), with the core operator (Fig. 4*A*) highlighted in *red*. DMBQ, dimethoxybenzoquinone.

Finally, we purified ^15^N-labeled WT SifR and subjected it to ^1^H,^15^N-transverse relaxation optimized spectroscopy (TROSY) (Fig. 9). The spectrum of the reduced SifR homodimer is of very poor quality and consistent with widespread intermediate conformational exchange that broadens all, but the sharpest cross peaks (likely from unstructured regions) beyond detection (Fig. 9, *upper left*). We next acquired a spectrum of SifR bound to the *fre* operator, which was selected for this experiment given identical half-sites (5-’TGTAA) and a nearly perfect palindromic between them, would minimize cross-peak doubling for those amide groups close to the DNA. Addition of stoichiometric *fre* DNA operator (23 bp; Fig. 4*A*) dampens this conformational exchange considerably, giving rise to significant chemical shift dispersion but still unassignable (Fig. 9, *upper right*). Subsequent addition of dimethoxybenzoquinone (DMBQ) gives rise to a spectrum that appears intermediate between the bound and free states (Fig. 9, *lower right*), but which compares well to the DMBQ-modified SifR free in solution (Fig. 9, *lower left*) as well as to the unmodified reduced SifR. We conclude that SifR, while conformationally dynamic, forms a high-affinity complex with operator DNA that is poised to rapidly react with an electrophile at C102, thus mediating DNA dissociation and transcriptional derepression.

## Discussion

**Figure 10:**
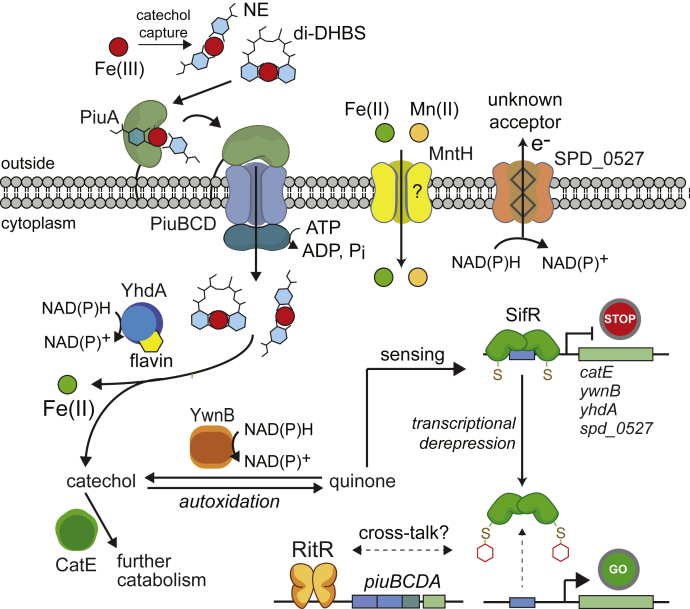
**Model for the SifR-regulated quinone detoxification system in *Streptococcus pneumoniae* D39 tied to Fe**^**III**^**–catecholate uptake through the PiuBCDA transporter** (25) **consistent with findings presented in this and prior work.** PiuA specifically binds coordinatively unsaturated tetradentate catecholate–Fe^III^ complexes (NE, norepinephrine; di-DHBS, an enterobactin hydrolysis product to which PiuA binds) (25), which are brought into the *S. pneumoniae* cytoplasm by PiuBCD. These complexes are processed by ferric reduction to Fe^II^ by YhdA, with the resulting catechol subjected to ring opening by CatE. Catechols quickly autoxidize to their corresponding quinones, which are re-reduced by the quinone reductase YwnB (and to a lesser extent by YhdA). Quinones are sensed by the quinone sensor SifR *via* thiol chemistry at C102, which induces DNA dissociation and transcriptional derepression of the SifR regulon. The function of SPD_0527, not defined here, may well function as an extracellular ferric reductase, which would allow Fe^II^ to enter the pneumococcal cell through MntH, whose expression is slightly increased in the Δ*sifR* strain. This is unknown at present. The presence of SifR also impacts *piuBCDA* expression, a known target of RitR (21), although this was not investigated here. CatE, catechol 2,3-dioxygenase; DHBS, 2,3-dihydroxybenzylserine.

In this work, we present the discovery and functional characterization of a new Rrf2-family transcriptional repressor in *S. pneumoniae* D39 that we denote SifR, encoded by *spd_1448*. We show that SifR is representative of a large SSN cluster (Fig. 2, cluster 6) and is the founding member of Rrf2-family monothiol quinone sensors, broadly conserved in streptococci and other Gram-positive Firmicutes, including Bacilli and Clostridia, and a few Actinobacteria (Fig. 2 and Table S1). We have defined the SIfR operator sequence and key functional features of the regulon that function to allow *S. pneumoniae* access to chemically diverse coordinately unsaturated Fe^III^–catecholate complexes, transported through PiuBCDA (25), in order to meet the needs for nutritional Fe, while avoiding the toxicity associated with catecholate-derived quinine-reactive electrophile chemistry (37) (Fig. 10). This is particularly important for *S. pneumoniae*, which is characterized by a comparatively small (≈2069 protein-encoding genes) genome (72) without the ability to synthesize its own siderophores, and thus is entirely dependent on Fe^III^ siderophores secreted by other microorganisms in the community and/or host-derived catecholamines. *S. pneumoniae* has evolved the capacity to bring Fe^III^–ferrichrome (a hydroxymate siderophore) complexes through the Pia transporter (73), while a more recent report describes a heme uptake system encoded by SPD_1590 (74). The systemic production of NE upon infection is a key feature of the antimicrobial response (31), and NE stimulates growth by helping to strip Fe from transferrin (25), which may well be a signal to the pneumococcus to disseminate to the lungs from the upper airway (2, 30). Consistent with this model, NE can be taken up by pneumococcal cells under these conditions, but it has not yet been established that this is absolutely dependent on PiuA (2). SifR is a virulence factor in the murine lung model of infection using a serotype 4 pneumococcal strain (75). As such, we propose from this work that virulence is further derived from the ability of *S. pneumoniae* to fine-tune the expression of genes controlling Fe^III^–catecholate assimilation during host infection.

We further document here that enzymes encoded by three of the most highly differentially expressed genes in a Δ*sifR* mutant possess the anticipated broad spectrum Fe^II^–catechol 2,3-dioxygenase activity (*spd_0072*; *catE*), quinone reductase activity (*spd_1440*; *ywnB*), and ferric reductase activity (*spd_1375*; *yhdA*) (76, 77). The first two activities in combination are expected to convert oxidized catechols, brought into the cytoplasm as Fe^III^ chelates, to the corresponding 2-hydroxy acid semialdehydes, whereas the third allows direct assimilation of ferrous Fe (Fig. 10). The extent to which each SifR-regulated gene product is required for Fe^III^–catecholate assimilation was not determined in this work, nor do we mean to imply an ordered pathway of Fe^III^ assimilation and quinone detoxification (Fig. 10). However, Tn-Seq screening shows that while CatE and YhdA are not essential for pneumococcal growth, a *catE* mutant is significantly attenuated in a murine lung model of infection, with the *yhdA* mutant somewhat less so (78). This is consistent with CatE and YhdA detoxification and assimilation, respectively, of host-provided catechol–Fe^III^ complexes (Fig. 10). These findings are in contrast to YwnB, where a *ywnB* mutant has no fitness defect in nasopharynx colonization or lung infection (78). This suggests the possibility that there may well be other quinone reductases that function in place of YwnB in a Δ*ywnB* strain, or YwnB has other biochemical activities not captured by these experiments.

One question left unanswered is the role of the integral membrane protein SPD_0527, which belongs to the diheme ferric reductase domain (56) or functionally analogous (79) superfamily of enzymes that are generally thought to reduce extracellular or periplasmic (in Gram-negative bacteria) Fe^III^ to Fe^II^ for import of Fe^II^ or an Fe^II^ complex across the plasma membrane (80). In the plant symbiont *Bradyrhizobium japonicum*, the enzyme analogous to SPD_0527 is FrcB, which is known to be under the transcriptional control of the global Fe regulator Irr but in this case is induced under conditions of low cellular Fe, as part of the Fe-scavenging response (58). *S. pneumoniae* lacks a characterized ferrous ion Feo-like transporter (80). However, it is known that a *S. pneumoniae* D39 triple mutant lacking the Piu, Pia, and Pit ABC transporters is still capable of obtaining Fe from a complex growth medium. This is consistent with the idea that other as-yet uncharacterized Fe acquisition systems exist in this organism, which may include SPD_1607-SPD_1609 and perhaps SPD_1590 (74, 81). A candidate ferrous ion importer is the NRAMP family Mn^II^/Fe^II^ transporter (82) MntH (SPD_0161; Fig. 10), which is uncharacterized in the pneumococcus, but functions as an infection-relevant Mn^II^ transporter in other streptococci and enterococci (83, 84, 85, 86). The expression of MntH is only slightly impacted by the loss of SifR (Fig. 3 and Table S5), which might suggest the possibility that MntH plays some role in response to changes in metal or catechol metabolism.

Ongoing studies are directed toward the identification and characterization of an NE sensing and degradation pathway in pneumococcal cells by leveraging an azido-NE derivative as a sole transferrin-derived Fe source; this will allow us to identify NE-interacting partners *via* implementation of a proteomics-based capture and enrichment strategy and may well identify new antimicrobial targets in this and related streptococcal pathogens. In addition, experiments are underway to elucidate the function of other gene products perturbed by the loss of SifR in *S. pneumoniae*, including the streptococci conserved operon (Fig. 3), which in some organisms is genomically linked to a gene encoding SifR.

## Experimental procedures

### SSN analysis

The EFI-EST (https://efi.igb.illinois.edu/efi-est/) web tools were used to generate SSNs using option A (sequence) with SPD_1448 and added InterPro Family IPR000944 as query to retrieve sequences. This resulted in the retrieval of 25,852 unique sequences in the UniRef90 dataset (79,708 accession IDs), which were then subjected to SSN cluster analysis using an alignment score (as) of 26 (sequences ≥40% identity will cluster into a single SSN cluster; trial 1) or 43 (sequences ≥50% identity will cluster; trial 2), with minimal and maximal sequence lengths of 100 and 200 residues, respectively. Final SSNs displayed and analyzed were 50% representative (repnode 50) for trial 1 (as 26) or 80% representative (repnode 80) for trial 2 (as 43), collapsing sequences of 50% or 80% identity over 80% of the sequence and visualized using Cytoscape 3.9 (http://www.cytoscape.org/) (87). Trial 2 was subjected to detailed analysis. The composite FASTA file containing all unique sequences associated with each SSN cluster was used to generate a multiple sequence alignment using Jalview (https://www.jalview.org) with sequences containing long N- and C-terminal extensions on either side of a core region, or those characterized by large insertions, removed to facilitate comparison of sequences within an SSN cluster. Multiple sequence alignments were then processed with CIAlign (88) to remove insertions for easier visualization prior to sequence logo generation by WebLogo 3 (89) that characterize each SSN cluster of interest. The list of sequences used to generate the logo plots (Fig. S3) is provided in Table S1*B*.

### Bacterial strain and plasmid construction

All primers are listed in Table S4. The Δ*sifR* mutant (IU10991) strain was constructed using standard laboratory practices for allelic replacement in WT *S. pneumoniae* serotype 2 D39W (IU1781) (Table S3). The various *sifR* repaired strains (WT, C84S, and C102S alleles) were constructed by allelic replacement in Δ*sifR* mutant (IU10991) with WT, C84S, and C102S *sifR* as amplicons. All constructs were sequence verified. For plasmid construction, genes encoding SifR (*spd_1448*), CatE (*spd_0072*), YhdA (*spd_1375*), and YwnB (*spd_1440*) were PCR amplified from *S. pneumoniae* D39 genomic DNA. Each gene was ligated into the pHis.parallel1 expression vector, transformed into *E. coli* DH5α, and selected for ampicillin resistance (100 μg/ml). Mutant plasmid alleles were prepared by PCR-based targeted site-directed mutagenesis using the parent expression plasmid as template. All plasmid constructs were sequence verified prior to transformation into *E. coli* BL21 (DE3) for protein expression and purification.

### RNA-Seq and qRT–PCR sample preparation and data analysis

Anaerobic growth experiments were performed in an anoxic chamber (85% N_2_, 10% H_2_, and 5% CO_2_) at 37 °C, whereas microaerophilic growth was conducted under an atmosphere of 5% CO_2_. For RNA-Seq, overnight exponential anaerobic *S. pneumoniae* cultures grown in brain heart infusion were diluted into prewarmed brain heart infusion to an absorbance of 0.005 at 620 nm, and growth was monitored over time. Cells were harvested at approximately an absorbance of 0.2 at 620 nm. Triplicate RNA samples were prepared for both WT and Δ*sifR* strains for RNA-Seq experiments. The RNA-Seq was preformed by the Center for Genomics and Bioinformatics at Indiana University, Bloomington. The RNA integrity number was determined with TapeStation (Agilent). The rRNA was removed using a Ribominus transcriptome isolation kit (Invitrogen; catalog no.: K1550), and a library was generated using a TruSeq stranded mRNA library prep kit (Illumina). The results of these experiments have been deposited in the Gene Expression Omnibus database under GenBank accession number GSE196501. Those genes with twofold change or greater in transcription level and BH-adjusted *p* value <0.05 were considered to be changed significantly. A similar RNA extraction procedure was followed for the qRT–PCR experiments, but cells were grown in microaerophilic conditions. Biological triplicate samples were prepared for each qRT–PCR experiment. The total RNA was extracted with the analysis carried out as described previously (90). PCR outcomes were normalized to the *gyrA* gene, and relative transcription levels were calculated by comparison of the ratio of mutant to WT.

### Protein expression and purification

For biochemical experiments, *E. coli* BL21 (DE3) containing target plasmids was grown in an LB medium supplemented with 100 μg/ml ampicillin at 37 °C. M9 minimal medium containing 100 μg/ml ampicillin and 1.0 g/l of ^15^NH_4_Cl (Cambridge Isotope Laboratories) as the sole nitrogen source was used to grow cells for NMR analysis. Protein expression was induced with 1 mM isopropyl β-d-1-thiogalactopyranoside at an absorbance of 0.8 at 600 nm and carried out overnight at 18 °C, after which cells were collected by centrifugation, cell pellets resuspended in buffer A (25 mM Tris–HCl, 500 mM NaCl, 2 mM TCEP, 10% glycerol, and 20 mM imidazole, pH 8.0), and lysed by sonication on ice. The crude lysate was clarified by centrifugation, followed by 70% ammonium sulfate precipitation. Precipitant containing target protein was resuspended in buffer A. Proteins were purified by Ni(II) affinity chromatography (GE Healthcare) with a concentration gradient moving from 100% buffer A to 100% buffer B (25 mM Tris–HCl, pH 8.0, 500 mM NaCl, 2 mM TCEP, 10% glycerol, and 500 mM imidazole). Pooled protein fractions were incubated with tobacco etch virus protease (20 μg/ml) to remove the hexahistidine tag during dialysis in buffer A containing 2 mM TCEP at 4 °C. Tag-free proteins were injected on to the Ni(II)-HisTrap FF column pre-equilibrated with buffer A. The flow through was collected and concentrated by centrifugation with a proper molecular weight cutoff filter before further separating proteins by size-exclusion chromatography using a Superdex-75 column pre-equilibrated with buffer C (25 mM Tris–HCl, pH 8.0, 500 mM NaCl, 2 mM EDTA, and 2 mM TCEP). Eluted proteins were pooled conservatively to obtain preparations of ≥95% purity as estimated by overloaded SDS-PAGE gels. The concentration of each purified protein was measured using the estimated molar extinction coefficient at 280 nm (ε_280_) of SifR (1490 M^−1^ cm^−1^), CatE (42,860 M^−1^ cm^−1^), YhdA (22,460 M^−1^ cm^−1^), and YwnB (12,950 M^−1^ cm^−1^). Purified proteins were routinely stored at −80 °C until use.

### Preparation of quinone-modified SifR protein mutants

All purified SifR protein mutants were buffer exchanged into degassed 50 mM Tris–HCl, 200 mM NaCl, 2 mM EDTA, pH 7.5 in an oxygen-free argon-filled glovebox (≤10 ppm O_2_) and diluted to 30 μM SifR dimer concentration. Freshly made 10 mM quinone stocks were prepared in the same buffer inside the glovebox. The buffer-exchanged proteins were reacted with a fivefold molar protein thiol excess of the indicated quinone compound for 1 h at ambient temperature. The remaining quinone was removed from the sample using a 3 kDa cutoff microconcentrator by centrifugation. The concentration of the modified SifR protein alleles was estimated using the Bradford assay since the quinone adduct impacts the ε_280_ value.

### Fluorescence anisotropy–based DNA–protein binding assays

The DNA oligonucleotides containing the SifR-binding site associated with each SifR regulon are listed in Table S4. The double-stranded 5′-fluorescein-labeled *catE* operator/promoter (O/P) DNA constructs were annealed as component single strands and titrated as previously described (60) using an ISS PC1 Spectrofluorometer equipped with an automatic titrator unit. About 10 nM fluorescein-labeled dsDNA in 25 mM Tris–HCl, 150 mM NaCl, 2 mM EDTA, 2 mM TCEP, pH 7.5 was titrated with SifR and SifR mutants with or without quinone modifications. The fluorescein was excited at 495 nm, and the polarization of the fluorescein fluorescence was monitored with a 515 nm cutoff filter in the L-format. Each data point collected was the average and standard deviation of three measurements. Normalized *r* values for the fractional saturation of *catE* O/P were calculated from (*r*_obs_ – *r*_0_)/(*r*_complex_ – r_0_) from 0 to 1 where *r*_complex_ represents the maximum anisotropy obtained and *r*_0_ represents free dsDNA. Collected data were fit to a nondissociable SifR dimer binding model using DynaFit (Biokin, Ltd) (91). Similar titrations were done using a competition assay, where protein was titrated into a mixture of fluorescein-labeled *catE* operator DNA, and the indicated unlabeled dsDNA duplex at 1:1, 3:1, or 5:1 molar ratio with the labeled DNA. The acquired data were fit to a nondissociable SifR dimer binding model using a global fitting script in DynaFit while fixing the *K*_*a*_ for the fluorescein-labeled *catE* DNA to its independently determined value (Table 1) and optimizing the *K*_*a*_ for the unlabeled duplex (Fig. S10). Simulations reveal that this approach can estimate a *K*_*a*_ ≤ ≈10-fold smaller than the *catE* O/P DNA, below which we obtain only an upper limit on *K*_*a*_.

Quinone modification–induced DNA–SifR dissociation experiments were carried out by monitoring the change in anisotropy upon direct addition of excess of quinone dissolved in same binding buffer without TCEP. The anisotropy of 10 nM *catE* O/P DNA was recorded for 150 s, after which time, stoichiometric (10 nM dimer) reduced WT or C84S SifR was added, and the anisotropy was recorded for 150 s. Quinones were added to a final concentration as 0.3 μM for BQ or 6 μM for Adc, and the anisotropy immediately recorded for another 150 s. Triplicate experiments were performed, and the raw anisotropy of a single representative experiment normalized as described previously.

### Catechol dioxygenase activity assay and product analysis

Purified *Sp*CatE was exchanged into oxygen-free reaction buffer of 25 mM Tris–HCl, 150 mM NaCl, pH 7.5 at a concentration of 500 μM protomer in the anaerobic glovebox. A freshly prepared Fe^II^ stock solution was made by dissolving ferrous ammonium sulfate in an oxygen-free reaction buffer. The *Sp*CatE was reactivated by addition of a 10-fold molar excess of Fe^II^ in the glovebox for 4 h, with unbound Fe^II^ removed with a 10 kDa cutoff spin column. CatE activity was assessed with various catechols as substrates in 100 mM phosphate buffer, pH 7.4, under ambient O_2_ and room temperature with 5 μM *Sp*CatE and 100 μM indicated catechol. The UV–Vis spectra of the reaction mixture were monitored continuously for 5 min, with the concentration of catechol cleavage products estimated by absorption at 375 nm with an extinction coefficient of 36,000 M^−1^ cm^−1^ (62). For analysis of *Sp*CatE-dependent degradation products, the enzyme was first activated as described previously, and 1 h reactions were carried out in 100 mM ammonium bicarbonate, pH 7.8, with 10 μM enzyme, 100 μM catechol, and 1 mM sodium ascorbate at 37 °C. The enzyme in these reactions was removed using a 3 kDa cutoff microfuge cartridge with the yellow-colored flow-through analyzed by high-resolution LC–MS.

### Ferric reductase activity and quinone reductase activity assays

*Sp*YhdA and *Sp*YwnB (0.5 μM) were evaluated for ferric reductase activity using 100 μM Fe^III^–NTA as the electron acceptor and NADPH or NADH (100 μM) and FMN or FAD (10 μM) as the reductant and electron donor, respectively (69). The reaction was carried out in 50 mM Tris–HCl, 150 mM NaCl, pH 7.5, 25 °C with 500 μM ferrozine. The chelation of Fe^II^ by ferrozine was monitored by the absorption at 562 nm at 2 min following addition of Fe^III^–NTA in the reaction. Freshly prepared 10 mM ferrous ammonium sulfate solution was serially diluted into the reaction buffer to generate a standard curve to quantify the Fe^II^ generated in each reaction. The ferric reductase activities of *Sp*YhdA and *Sp*YwnB with Fe^III^–di-DHBS (50 μM) and Fe^III^–NE (50 μM) complexes as electron acceptor were prepared as described previously (25).

The quinone reductase activities of *Sp*YhdA and *Sp*YwnB were evaluated with 0.1 mM BQ or 0.1 mM Adc as electron acceptors and 10 μM FMN (for *Sp*YhdA assay only), 100 μM NAD(P)H as reductant and electron donor, respectively, in 50 mM Tris–HCl, 150 mM NaCl, pH 7.5 for 5 min at room temperature. The quinone reductase activity was evaluated by consumption of NADPH at 340 nm over time and quantified using an extinction coefficient of 6200 M^−1^ cm^−1^. The background reaction rate without addition of enzyme was also monitored and subtracted from the enzyme-containing reaction prior to data analysis. The averaged reductase activities were quantified as nmol Fe^II^ generated (ferric reductase) or NAD(P)H consumed (quinone reductase) per minute per milligram enzyme in these single time-point assays.

### Ratiometric pulsed-alkylation MS analysis

Sample preparation for pulsed-alkylation MS was adapted from a previous report and optimized for SifR (59). All experiments were carried out anaerobically in a glovebox in a buffer containing 10 mM Hepes and 200 mM NaCl at pH 7.0. WT and C84S SifRs with or without 1.5 M excess of 30 bp *S. pneumoniae catE* SifR O/P dsDNA oligo was reacted with a threefold molar thiol excess of *d*_5_-NEM (*pulse*, Isotech). At discrete time points, 50 μl aliquots were withdrawn and quenched with an equal volume of a solution containing a 900-fold thiol excess of H_5_-NEM (*chase*) with 100 mM Tris (pH 8.0) and 8 M urea. After a 40 min chase, quenched reactions were removed from the glovebox and precipitated on ice with a final concentration of 12.5% trichloroacetic acid for 1.5 h. Precipitated protein was pelleted by centrifugation at 4 ˚C. The supernatant was removed, and the pellet was washed twice with ice-cold acetone. The washed pellet was vacuum centrifuged to dryness at 45 ˚C and resuspended in 10 μl digestion buffer (20 mM ammonium bicarbonate, 10% acetonitrile, 50:1 protein:trypsin ratio, pH 8.2) for 30 min at 37 °C. Tryptic digests were quenched with a final concentration of 1% TFA and spotted on a MALDI plate with α-cyano-4-hydroxycinnamic acid matrix using a 5:1 matrix:sample (v/v) ratio for this analysis.

MALDI-TOF mass spectra were collected and analyzed in triplicate reactions using a Bruker Autoflex III MALDI-TOF mass spectrometer with 200 Hz frequency-tripled Nd:YAG laser (355 nm) and Flex Analysis software (Bruker Daltonics). Cysteine-containing peaks were identified by their corresponding monoisotopic masses (Table S5) and resolved as alkylated with *d*_5_-NEM (+130.0791 Da) or H_5_-NEM (+125.0477 Da) with little to no detectable unmodified peptide detected under these conditions (data not shown). The theoretical distribution and peak areas were determined using the averaging algorithm (38) and quantified by summing the total peak areas of the full isotopic distribution. Relative peak areas were used to determine the mole fraction of H_5_-NEM–labeled peptide, Θ(H_5_), as defined by Equation 1. A(H_5_) and A(*d*_5_) correspond to the area (A) of the isotopic distribution of H_5_-NEM or *d*_5_-NEM alkylated peptide, respectively. To obtain the pseudo–first-order rate constant of alkylation, *k*, Θ(H_5_) was plotted as a function of pulse time, *t*, and fit to Equation 2. In some instances, a fit to a sum of two exponentials was used, Equation 3. The second-order rate constant was obtained by dividing *k* by the concentration of *d*_5_-NEM in the pulse.

(1) $$\Theta \left(H_{5}\right)=\frac{A\left(H_{5}\right)}{A\left(H_{5}\right)+A\left(d_{5}\right)}$$

(2) $$\Theta \left(H_{5}\right)=\Theta {\left(H_{5}\right)}_{t0}•e^{-kt}$$

(3) $$\Theta \left(H_{5}\right)=\Theta {{\left(H_{5}\right)}_{t_{0}}}_{-t_{\text{slow}}}•e^{-k_{\text{fast}}t}+\Theta {\left(H_{5}\right)}_{t_{0}-t_{\text{fast}}}•e^{-k_{\text{slow}}t}$$

### Protein LC–MS and LC–MS/MS

The reduced WT and C84S SifR proteins were reacted with a 20-fold molar excess of 1,4-BQ or Adc in 25 mM Tris–HCl, 150 mM NaCl, 2 mM EDTA, pH 7.5, for 1 h at room temperature. The ESI–MS spectrum of reduced and quinone-modified protein was recorded using an LC (C4 reverse phase)–MS (Synapt G2S HDMS) instrument. Mass spectra were analyzed using MassLynx, version 4.1 (Waters, Inc) and OriginPro 2018 (Origin Lab, Inc). The quinine-modified SifR WT and SifR C84S were digested by trypsin following the same protocol as the aforementioned MALDI-TOF sample preparation, and the peptides were fragmented and characterized by a Thermo Scientific Orbitrap Fusion LUMOS instrument. Peptides containing C102 with a 1.4-BQ adduct (+106.01 Da quinone state, +108.02 Da hydroquinone state) or an Adc adduct (+177.04 Da quinone state, +179.06 Da hydroquinone state) were used to query the corresponding LC–MS/MS spectra.

### Protein NMR spectroscopy

NMR samples contained 200 μM SifR (protomer) in various allosteric states, with 25 mM MES (pH 6.5), 150 mM NaCl, and 10% v/v D_2_O, with 0.3 mM 2,2-dimethyl-2-silapentanesulfonic acid as an internal reference. The protein–DNA complex sample contained a slight molar excess of the nearly palindromic 23-bp *fre* DNA operator (1:1) to ensure a similar chemical environment for both SifR protomers and minimize the likelihood of different chemical shifts for the same residue. The quinone-modified protein–DNA sample was generated by adding 400 μM DMBQ directly to the complex. A fourth sample contained 200 μM SifR modified with 400 μM DMBQ. ^15^N,^1^H transverse relaxation optimized spectroscopy spectra were recorded at 25 °C on a Bruker Avance Neo 600 MHz spectrometer equipped with a cryogenic probe in the METACyt Biomolecular NMR Laboratory. Data were collected, processed, and analyzed as described in previous work (25).

## Data availability

All data described in the article are contained within the article, with the RNA-Seq data deposited at https://www.ncbi.nlm.nih.gov/geo/ under accession number GSE196501. The SifR structural model is available in ModelArchive at https://modelarchive.org/doi/10.5452/ma-6pz9c. The *Sp*YhdA structural model is available in ModelArchive at https://www.modelarchive.org/doi/10.5452/ma-2regv.

## Supporting information

This article contains supporting information (72).

## Conflict of interest

The authors declare that they have no conflicts of interest with the contents of this article.
