# Supplementary material for: SifR is an Rrf2-family quinone sensor associated with catechol iron uptake in Streptococcus pneumoniae D39
Source: J Biol Chem. 2022 May 18;298(7):102046. doi: 10.1016/j.jbc.2022.102046 (PMC9218516; doi:10.1016/j.jbc.2022.102046)
Supplement: Supplemental Tables S3, S4, S6–S8 and Figs. S1–S10 [file mmc3.pdf]

*Supporting Information*

# SifR is an Rrf2-family quinone sensor associated with catechol iron uptake in *Streptococcus pneumoniae* D39

Yifan Zhang[1,2], Julia E. Martin[1,3], Katherine A. Edmonds[1], Malcolm E. Winkler[2,4] and David P. Giedroc[1,2]

[1]Department of Chemistry, Indiana University, Bloomington, IN 47405-7102 USA
[2]Department of Molecular and Cellular Biochemistry, Indiana University, Bloomington, IN 47405 USA
[3]Department of Biological Sciences, Idaho State University, Pocatello, ID 83209 USA
[4]Department of Biology, Indiana University, Bloomington, IN 47405 USA

This file contains Supplementary Tables S1-S8 and Supplementary Figures S1-S10.

**Supplementary Table S1.** All UniProt sequences (UniRef90) that comprise the entire SSN analysis shown in Fig. 2, main text, sorted by sequence count cluster number (column D, the unique SSN cluster number used here throughout), with a secondary sort on the number of the IDs per node (column I). Each node contains UnieRef90 sequence that are 80% identical over 80% of the sequence. This is a searchable Excel file using UniProt ID, locus tag, PDB code or organism as query for example, exported from Cytoscape.

**Supplementary Table S2**. List of UniProt IDs from the SSN analysis (Fig. 2, main text) highlighting Rrf2 family proteins found in clusters 1-10, 13, 15, 20 and 43 discussed in the text, grouped on sheets 1-14, respectively. This is searchable Excel file exported from Cytoscape. WebLogo plots of sequence conservation for each of these clusters are shown in Fig. S3.

**Supplementary Table S3.** Bacterial strains and plasmids used in this study.

| **Strain** | **Genotype** | **Reference** |
|---|---|---|
| ***S. pneumoniae*** | | |
| IU1781 | D39W *rpsL1* ($Str^R$) | (72) |
| IU10991 | D39W *rpsL1* Δ*sifR*::[$Kan^R$ *rpsL*$^+$] | This work |
| IU18110 | D39W *rpsL1* sifR C84S | This work |
| IU18112 | D39W *rpsL1* sifR C102S | This work |
| IU18790 | D39W *rpsL1* sifR repaired | This work |
| ***E. coli*** | | |
| DH5α | $F^-$ endA1 *glnV44 thi-1 recA1 relA1 gyrA96 deoR nupG purB20* φ80d*lac*ZΔM15 Δ(*lacZYA-argF*)U169 hsdR17($r_K^-$ $m_K^+$), $λ^-$ | Lab stock |
| BL21 | $F^-$ *omp*T *hsd*$S_B$ ($r_B^-$, $m_B^-$) *gal dcm* (DE3) | Lab stock |
| **Plasmid** | **Relevant Characteristics** | **Reference** |
| pHis-Parallel1 | Protein expression vector, $Amp^R$ | Lab stock |
| pSifR | pHis.Parallel1 with *sifR* (*spd_1448*) | This work |
| pSifR-C84S | pHis.Parallel1 with *sifR*-C84S | This work |
| pSifR-C102S | pHis.Parallel1 with *sifR*-C102S | This work |
| pSifR-DM | pHis.Parallel1 with *sifR*-C102S/C84S | This work |
| pCatE | pHis.Parallel1 with *catE* (*spd_0072*) | This work |
| pYhdA | pHis.Parallel1 with *yhdA* (*spd_1375*) | This work |
| pYwnB | pHis.Parallel1 with *ywnB* (*spd_1440*) | This work |

**Supplementary Table S4.** DNA oligos used in this study.

| Oligo | Sequence 5' to 3' |
|---|---|
| **Construction of *S. pneumoniae* ΔsifR** | |
| FP_outside_*sifR* | CTTTCAGATATTCATGGAAATACCACCGCC |
| RP_outside_*sifR* | CTCCATCAAGAATTGACACAGACTGCGG |
| RP_*sifR*_kanrpsL_5' | CATTAAAAATCAAACGGATCCTACTCGAGGGCAATGATTATCAGCATATG |
| FP_*sifR*_kanrpsL_5' | CTGATAATCATTGCCCTCGAGTAGGATCCGTTTGATTTTTAATGGATAATG |
| RP_*sifR*_kanrpsL_3' | CACGACTTTTTCTAGACTGGTGGGCCCCTTTCCTTATGCTTTTGG |
| FP_*sifR*_kanrpsL_3' | AAGCATAAGGAAAGGGGCCCACCAGTCTAGAAAAAGTCGTGG |
| SifR_C102S_FP | GATGTTTATCAGGCGGTCGAAAGTCTTGGTAAGACAGGTCAACTCTTC |
| SifR_C102S_RP | CCATGACAATCCGAATCCAAATAGCCCTGTAGGAGCTCATATTCATG |
| SifR_C84S_FP | GATGTTTATCAGGCGGTCGAAAGTCTTGGTAAGACAGGTCAACTCTTC |
| SifR_C84S_RP | GAAGAGTTGACCTGTCTTACCAAGACTTTCGACCGCCTGATAAACATC |
| **Construction of expression plasmids** | |
| *Sp*SifR_pHis_FP | AAACCTGTATTTTCAGGGCGCCATGGGTCAAATTCCAAGTAGATTTACCATTGCAACT |
| *Sp*SifR_pHis_RP | GTGGTGCTCGAGTGCGGCCGCAAGCTTTTAATCCTTCATCTGACTCTCTGCATCG |
| *Sp*YhdA_pHis_FP | AAACCTGTATTTTCAGGGCGCCATGGGTTCTAAAAAAGTATTATTTATCGTCGGATC |
| *Sp*YhdA_pHis_RP | GTGGTGCTCGAGTGCGGCCGCAAGCTTTTACTGAATCGCTTCGACC |
| *Sp*YwnB_pHis_FP | AAACCTGTATTTTCAGGGCGCCATGGGTAAACTAGCAGTTATTGCAGCAAATGG |
| *Sp*YwnB_pHis_RP | GTGGTGCTCGAGTGCGGCCGCAAGCTTTTATTTTTCAAGTAAAGAGATACGTTC |
| SpCatE_pHis_FP | AAACCTGTATTTTCAGGGCGCCATGGGTACCTATGAATATAAGAGTCACATTTATTTGG |
| *Sp*CatE_pHis_RP | GTGGTGCTCGAGTGCGGCCGCAAGCTTTTACCTTGCTAAACGAATACGGGTCAC |
| *Sp*SifR_C84S_FP | GATGTTTATCAGGCGGTCGAAAGTCTTGGTAAGACAGGTCAACTCTTC |
| *Sp*SifR_C84S_RP | GAAGAGTTGACCTGTCTTACCAAGACTTTCGACCGCCTGATAAACATC |
| *Sp*SifR_C102S_FP | CCATGACAATCCGAATCCAAATAGCCCTGTAGGAGCTCATATTCATG |
| *Sp*SifR_C102S_RP | CATGAATATGAGCTCCTACAGGGCTATTTGGATTCGGATTGTCATGG |
| **qRT-PCR** | |
| *catE*_FP | CCTGGCAGAAGGTACAAGAAT |
| *catE*_RP | TATCCTCTAGCCCTAACACCTT |
| *yhdA*_FP | CCTGCATTGGCTACAGATGA |
| *yhdA*_RP | ACTTGCTTGACTGGCTATCTC |
| *ywnB*_FP | ATGCTGTTATTTCAGCGTTTGG |
| *ywnB*_RP | TGTCTGTGTTCCAGCAAGAAG |
| *spd_0527*_FP | CCATCTTAGCAGACCTTTCAACTA |
| *spd_0527*_RP | GACCATGACCTCCTGAGATAGA |
| *trxB*_FP | GCCTTCGGTGGTGTCTTTAT |
| *trxB*_RP | GCAGTCTTCATGTGGCTATCT |

| DNA anisotropy titrations | |
|---|---|
| *catE*_O/P | /Fluorescein dT/ATAGTTGTAATAATAATAATTACAACGAG |
| | CTCGTTGTAATTATTATTATTACAACTATA |
| *yhdA*_O/P | GTTTTGTTGTAATATATTTTATAACAACGAG |
| | CTCGTTGTTATAAAATATATTACAACAAAAC |
| *ywnB*_O/P | CAATTGCTGTAACTAAAATAATTACAACAAG |
| | CTTGTTGTAATTATTTTAGTTACAGCAATTG |
| *trxB*_O/P | GAAAGAGTTAGAATAAAGATAGTTACAACAAG |
| | CTTGTTGTAACTATCTTTATTCTAACTCTTTC |
| *fre*_O/P | GATAAAATGTAACAAATTTATTTACAACAAATC |
| | GATTTGTTGTAAATAAATTTGTTACATTTTATC |

**Supplementary Table S5.** Excel file that documents all transcriptomic changes in the Δ*sifR* vs. the wild-type strain using RNAseq (anaerobic growth conditions). A) Change in expression of selected genes; B) Change in expression of all genes.

**Supplementary Table S6.** Peptide mass list for ratiometric pulsed-chase alkylation-mass spectrometry analysis

| Peptide | Amino acid sequence | Mass (Da) | Cys[a] | Modification | Mass of modified peptides (Da) Calculated | Observed |
|---|---|---|---|---|---|---|
| 88-118 | TGQLFSFHDNPNPNC PVGAHIHDVLDQKLER | 3498.7 | C102 | $H_5$-NEM | 3623.7 | 3623.8 |
| | | | | $d_5$-NEM | 3628.7 | 3628.8 |

[a]Peptide containing C84 is not detected likely due to poor ionization efficiency.

**Supplementary Table S7.** Protein masses obtained from LC-MS analysis of derivatized SifR

| | | | Mass of modified proteins (Da) | |
|---|---|---|---|---|
| Protein | Cys[a] | Quinone Modification | Calculated[a] | Observed[b] |
| SifR$^{WT}$ | C84, C102 | *p*-Benzoquinone | 16226.86 | 16227 |
| | | Adrenochrome | 16189.84 | 16186 |
| | | No treatment | 16010.67 | 16011 |
| SifR$^{C84S}$ | C102 | *p*-Benzoquinone | 16103.56 | 16100 |
| | | Adrenochrome | 16173.78 | 16173 |
| | | No treatment | 15994.61 | 15994 |

[a]The calculated mass of direct quinone modification without further oxidation on cysteine quinone adducts. [b]Only the major modified peaks are shown. Putative multiple adrenochrome adduction peaks not shown (see Fig. 8).

**Supplementary Table S8.** Observed and expected masses of the *Sp*CatE substrates and products

| Substrate | Mass of Substrate (Da) | Mass of Products (Da) | |
|---|---|---|---|
| | | Calculated | Observed |
| Catechol | 110.04 | 142.03 | 141.0175 $[M-H]^-$ |
| DHBS | 241.06 | 273.05 | 272.0399 $[M-H]^-$ |
| Epinephrine (EP) | 183.09 | 215.08 | 216.0937 $[M+H]^+$ |
| Norepinephrine (NE) | 169.07 | 201.06 | 202.0760 $[M+H]^+$ |

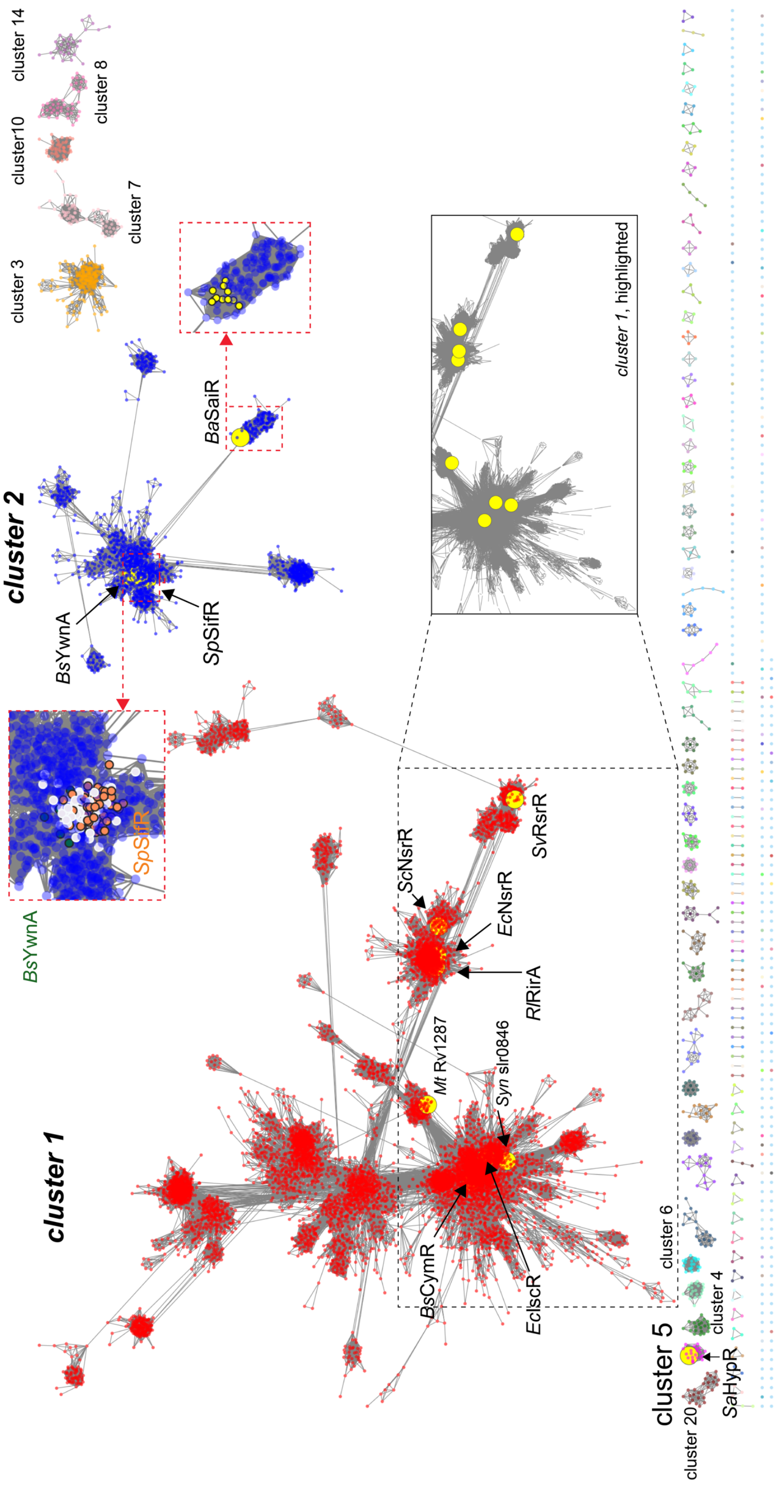


**Supplementary Figure S1**. (A) Sequence similarity network (SSN) analysis of Rrf2 superfamily of transcriptional regulators using SPD_1448 as query (InterPro Family: IPR000944) with an alignment score of 26 (sequences greater than 40% identity are grouped in individual SSN clusters). The entire network is shown and are ranked according to the number of unique sequences in each SSN cluster (with 1 being the largest number of sequences) and arranged

from *upper left* to *lower right* by decreasing numbers of sequence nodes (each node contains sequences that are 50% identical over 80% of the sequence). SSN cluster 2 (node cluster rank 2) harbors *Sp*SifR characterized in this work. Expanded regions of SSN cluster 2 highlight the degree to which SifR (*orange*), YwnA (*forest green*) and other monothiol Rrf2 sequences (*light purple;* SSN cluster 4) from the SSN analysis of Fig. 2 (main text) are related. *Bs*SaiR (cluster 15, Fig. 2) defines its own cluster 2 sub-cluster, while *Sa*HypR (cluster 43, Fig. 2) is segregated even here. *Ec*, *E. coli*; *Bs*, *B. subtilis*; *Ba*, *B. anthracis*; *Sc*, *Streptomyces coelicolor*; *Syn*, *Synechocystis*; *Rl*, *Rhizobium leguminoserum*; *Sv*, *Streptomyces venezuelae*; *Mt*, *Mycobacterium tuberculosis*; *Sa*, *Staphylococcus aureus*. See text for other details.

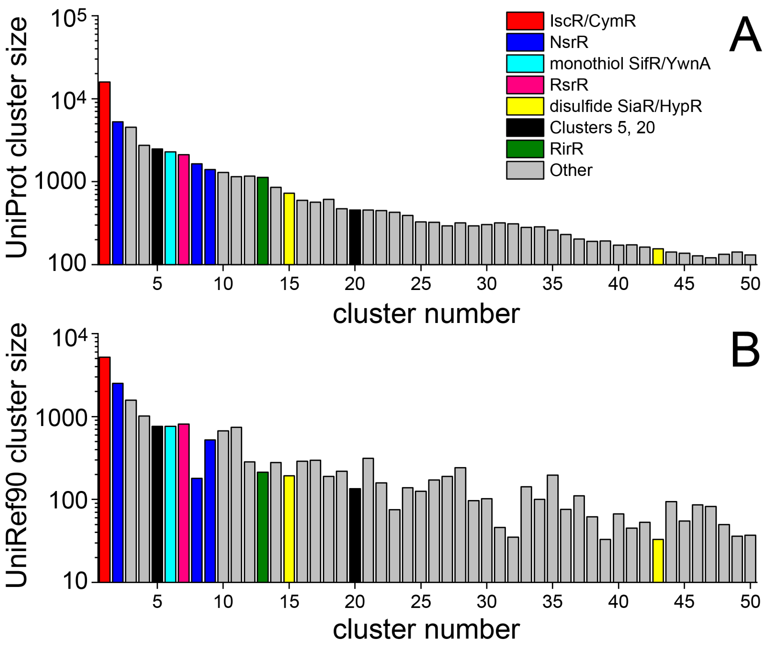


**Supplementary Figure S2.** Bar chart representation of the distribution of sequences per SSN cluster for clusters 1-50, ranked according to (A) total number of sequence and (B) total number of UniRef90 sequences from highest (cluster 1) to lowest (cluster 50) (from the analysis shown in Fig. 2, main text).

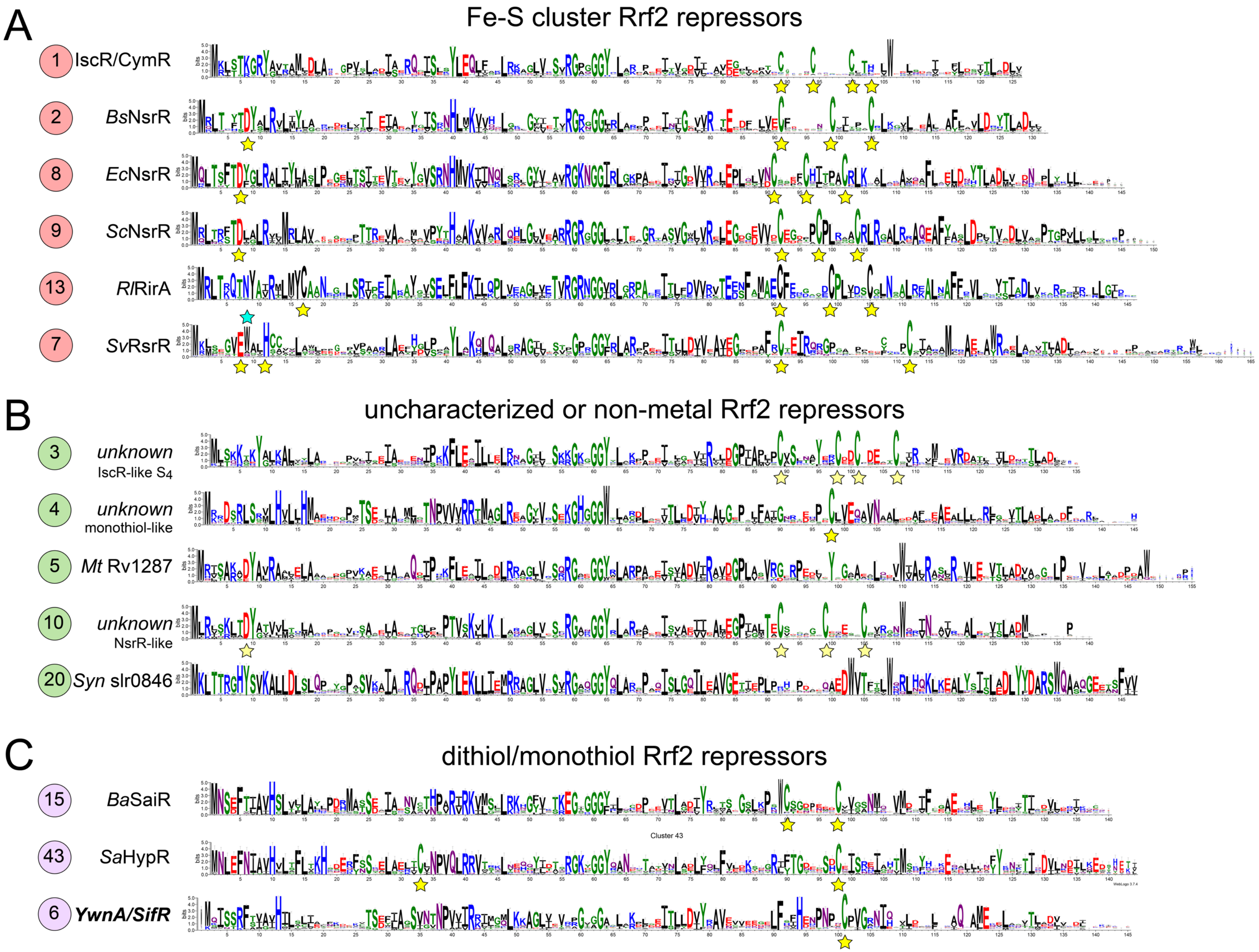
A
Fe-S cluster Rrf2 repressors
1 IscR/CymR
2 BsNsrR
8 EcNsrR
9 ScNsrR
13 RlRirA
7 SvRsrR
B
uncharacterized or non-metal Rrf2 repressors
3 unknown IscR-like S4
4 unknown monothiol-like
5 Mt Rv1287
10 unknown NsrR-like
20 Syn slr0846
C
dithiol/monothiol Rrf2 repressors
15 BaSaiR
Cluster 43
43 SaHypR
6 YwnA/SifR

**Supplementary Figure S3.** WebLogo plots that illustrate amino acid sequence conservation in the SSN clusters corresponding to representative SSN clusters in Fig. 2 (main text; alignment score of 43). These are organized from top to bottom in order of discussion in the text, with the "wing-tip" RGxxGGY sequence vertically aligned (see text for details). (A) Characterized Fe-S cluster containing Rrf2 repressor sequences derived from SSN clusters 1, 2, 8, 9, 13 and 7, with the SwissProt-curated repressor names indicated where known. (B) Biochemically uncharacterized Rrf2 repressor sequences derived from large SSN clusters 3, 4, and 10 and SwissProt-curated repressors from cluster 5 and 20, which appear to lack metal-coordinating residues. (C) Dithiol and monothiol Rrf2-family repressors from SSN clusters 15, 43 and 6. *Yellow* stars, known Fe-S cluster ligating residues or cysteines that participate in the disulfide or electrophile chemistry. *Light yellow* stars in panel B correspond to candidate regulatory residues. All sequences and associated UniProt numbers for each WebLogo representation are compiled in Table S2.

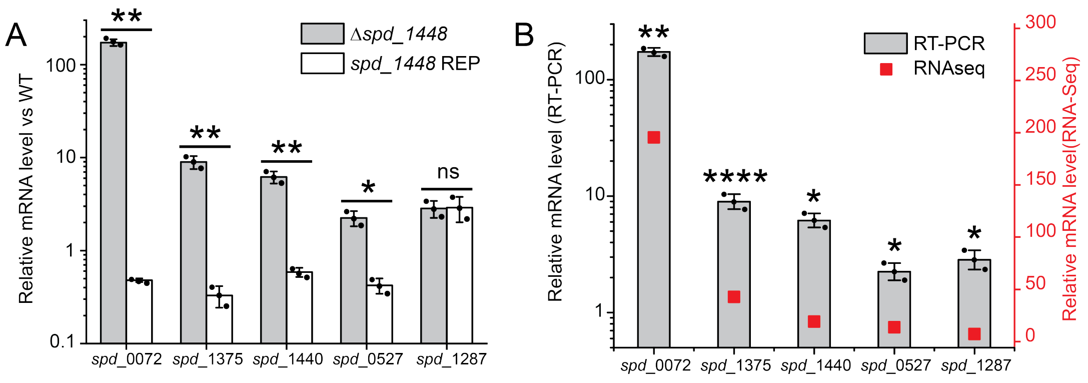


**Supplementary Figure S4**. (A). qRT-PCR analysis of candidate SifR regulated genes in Δ*spd_1448* strain (IU10991) vs. *spd_1448* repaired (REP) strain (IU18790) (Table S1). (B). qRT-PCR analysis of the differences in gene expression in Δ*spd_1448* strain (IU10991) measured under standard microaerophilic conditions (bars) from triplicate cultures vs. aerobic conditions by RNA-seq (*red* squares) (for a complete list of genes, see Table S5, Fig. 3). * *P*<0.05, ** *P*<0.01, *** *P*<0.001, **** *P*<0.0001. Error bars represent the SD from biological triplicate measurements, with each data point also shown (*black* circles).

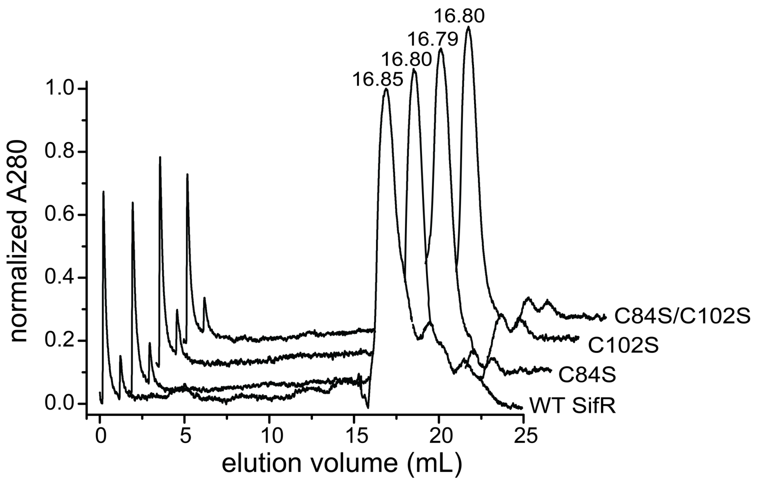


**Supplementary Figure S5.** Gel filtration chromatography of wild-type and mutant SifR proteins used in this work. Buffer conditions, 50 mM Tris-HCl, 150 mM NaCl, 2mM EDTA, 2 mM TCEP pH 8.0, 10 μM protomer concentration (5 μM dimer).

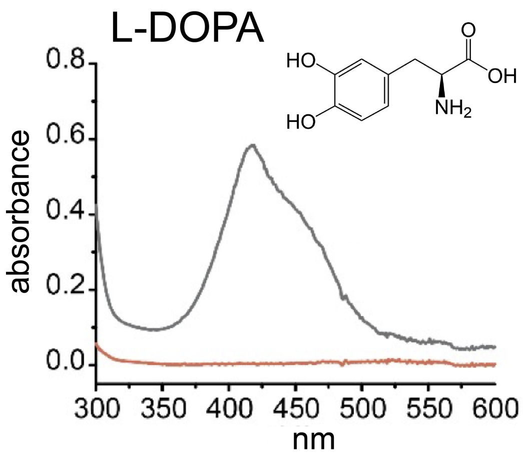


**Supplementary Figure S6.** Electronic absorption spectra (*red*, substrate; *black*, product) of the product of CatE-catalyzed cleavage of L-DOPA (5 min). Conditions: 5 μM $Fe^{II}$-CatE, 100 μM indicated catechol, pH 7.4, ambient temperature.

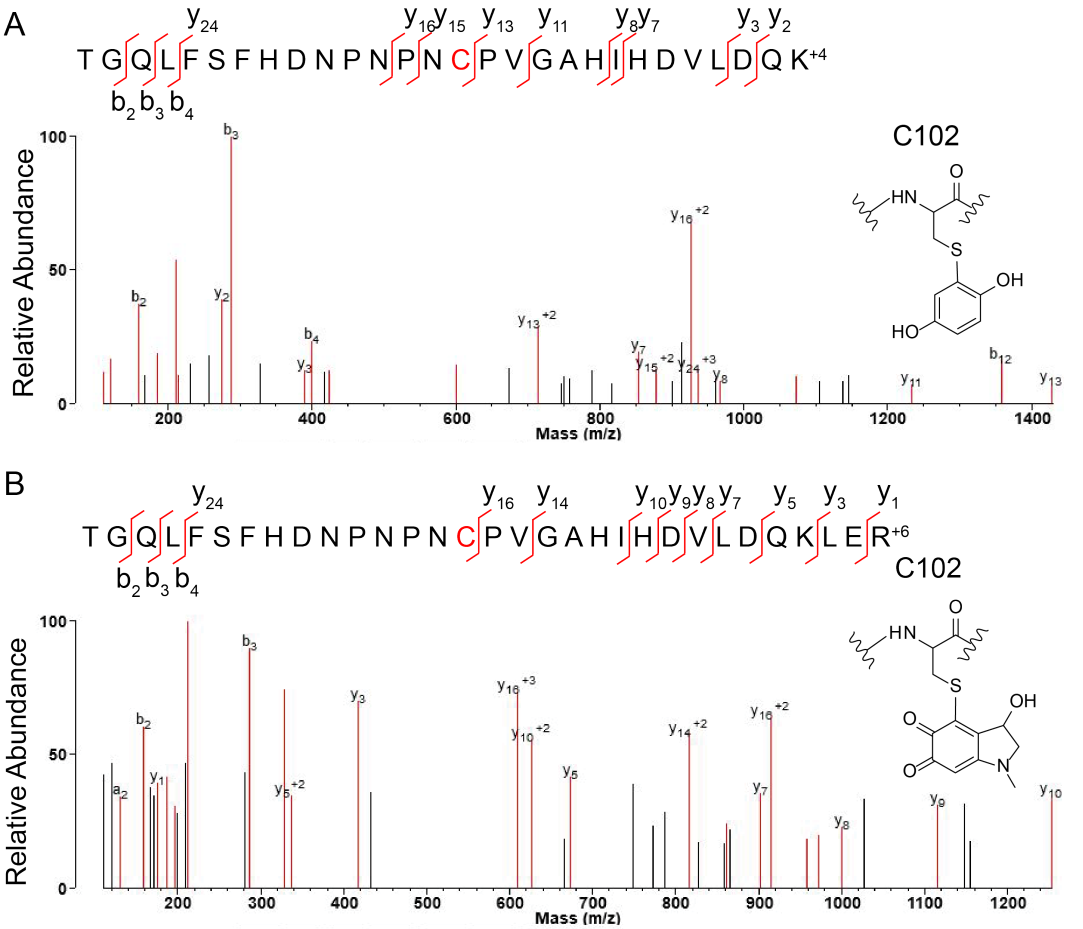


**Supplementary Figure S7.** Fragmentation and characterization of peptides with (A) 1,4-benozoquinone and (B) adrenochrome adducts at C102 from WT SifR WT or C84S SifR, respectively, by LC-MS/MS. The peptide containing C84 is not detected, likely due to low ionization efficiencies.

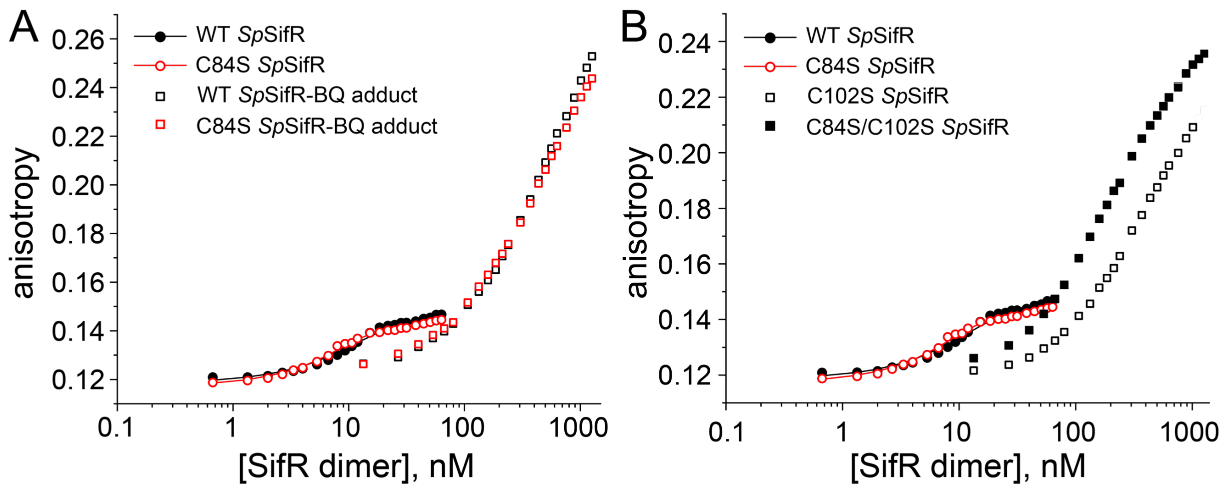


**Supplementary Figure S8.** (A) Representative *catE* O/P fluorescence anisotropy-based binding isotherms obtained for benzoquinone (BQ)-derivatized WT SifR (*black*) and C84S SifR (*red*) compared to corresponding unmodified and reduced SifR. The binding curves obtained for the quinone-modified SifR are consistent with non-specific binding to the DNA that is not saturable. (B) Analogous binding isotherms obtained for SifR proteins containing any substitution of C102. The binding curves obtained for the C102-substituted SifR are consistent with non-specific binding to the DNA that is not saturable. Conditions: 50 mM Tris-HCl, 150 mM NaCl, pH 7.4, 25.0 ºC.

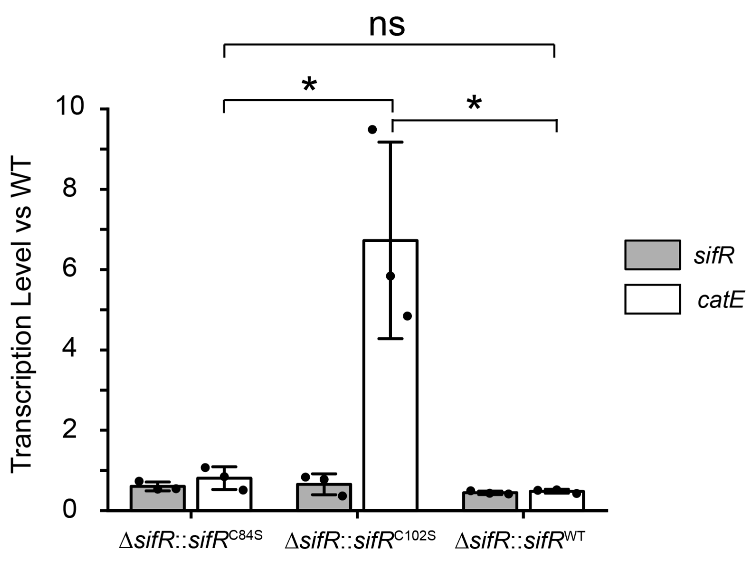


**Supplementary Figure S9**. qRT-PCR analysis of the differences in the expression of the *sifR (filled bars)* and *catE* (*open* bars) genes in the following repaired *S. pneumoniae* D39 allelic replacement strains: Δ*sifR*::*sifR*$^{C84S}$, Δ*sifR*::*sifR*$^{C102S}$ and Δ*sifR*::*sifR*$^{WT}$ measured under standard microaerophilic conditions from biological triplicate cultures. The gene expression level is normalized to expression of the *sifR* gene in the wild-type strain (IU1781) under the same

growth conditions. * $P$<0.05. Error bars represent the SD of biological triplicate samples, with the individual data points shown (*black* circles).

```
________________________________________________________________________
Competitive fluorescence displacement assay.
P = protein;
D = catE DNA;
C = Compet DNA;
Objective: determine both Kd's,
including confidence intervals.
;
_______________________________________________________________________
[task]


data = equilibria

task = fit

[mechanism]

P + D <==> PD   :    K1   assoc.

P + C <==> PC   :    K2   assoc.

[constants]
   K1 = 0.102 ; nM-1                              #Known binding affinity for catE O/P DNA
   K2 = 0.025? ; nM-1                             #Unknown binding affinity for compet O/P DNA

[data]

directory                               #Directory of Titration datafile  (protein conc. vs anisotropy)
extension txt



variable P
file DataFile_1 | response D = 0.0134? , PD = 0.0168? , C = 0 , PC = 0 | conc. D = 10 , C = 10
file DataFile_2 | response D = 0.0130? , PD = 0.0165? , C = 0 , PC = 0 | conc. D = 10 , C = 30
file DataFile_3 | response D = 0.0135? , PD = 0.0170? , C = 0 , PC = 0 | conc. D = 10 , C = 50

[output]

directory                               #Directory of Output file

[end]
;
```

**Supplementary Figure S10.** Dynafit script used to globally analyze the fluorescence anisotropy-based DNA binding competition experiments (see Fig. 4, main text).
